# Low-density lipoproteins in human serum competitively inhibit the binding and entry of vesicular stomatitis virus

**DOI:** 10.1016/j.omta.2026.201721

**Published:** 2026-03-19

**Authors:** Rianna Vandergaast, Samantha Johnson, Christopher Ziegler, Gopal Naik Nenavath, Luke Schnebeck, Riya Narjari, Stephen J. Russell

**Affiliations:** 1 Imanis Life Sciences, LLC, Rochester, MN, USA; 2 Vyriad, Inc, Rochester, MN, USA

**Keywords:** serum, lipoproteins, LDLR, inhibition, LDL, VLDL, VSV-G, retargeted, *in vivo* vector delivery

## Abstract

Vesicular stomatitis virus (VSV) is a promising anticancer agent but rapidly loses its infectivity in human serum. Here, we demonstrate that low-density and very low-density lipoproteins (LDLs and VLDLs) compete with VSV-G-displaying vectors for occupancy of cellular LDL receptors (LDLRs). Infectivity of VSV-G-displaying vectors was reduced two- to three-logs in the presence of heat-inactivated human serum but not if the serum was depleted of lipoproteins. The inhibitory effect was replicated in serum-free media supplemented with physiological concentrations of purified lipoprotein particles that contain the LDLR-binding protein Apo B-100, and the degree of inhibition correlated with the concentration of LDL or VLDL. When VSV-G was retargeted to the epidermal growth factor receptor, competitive inhibition by human serum or by LDL/VLDL was no longer observed. Similar results were obtained when VSV-G was retargeted to other receptors. Our findings demonstrate that serum LDL and VLDL competitively block the entry of VSV-G-displaying viral particles, but this barrier for *in vivo* delivery can be circumvented through a display of a receptor-targeting ligand on VSV-G.

## Graphical abstract

**Figure:**
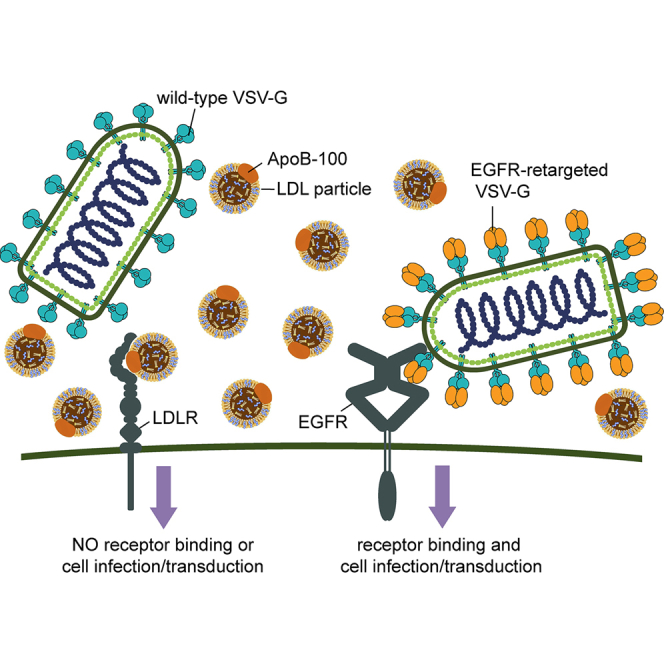

## Teaser

Vandergaast and colleagues demonstrate that serum lipoproteins (LDL and VLDL) competitively inhibit VSV- and VSV-G-displaying vectors from binding to LDL receptors and entering cells. Retargeting VSV-G to a non-LDL receptor rescues vector infectivity, highlighting the importance of retargeting VSV-G for *in vivo* vector delivery applications.

## Introduction

Replicating viruses and viral vectors are being developed as therapeutics to treat a wide range of diseases from cancer to genetic disorders. One of these viruses, recombinant Indiana strain vesicular stomatitis virus (VSV), is currently in phase 1 and 2 clinical trials as a potential oncolytic therapeutic.^1^^,^^2^^,^^3^ Binding and entry of VSV are governed by its homotrimeric membrane glycoprotein, VSV-G, which is also frequently used to pseudotype lentiviral vectors (LVs). Although there has been considerable recent progress re-engineering the tropisms of VSV and VSV-pseudotyped vectors to target specific cell populations,^4^^,^^5^^,^^6^^,^^7^ a major potential roadblock to their clinical translation has been the high efficiency with which human serum is known to inactivate them.

VSV-G is incorporated into the membranes of budding vector particles in a closed or pre-fusion conformation.^8^^,^^9^^,^^10^ In its pre-fusion form, VSV-G binds to the cysteine-rich repeat (CR) domains CR2 and CR3 of the low-density lipoprotein receptor (LDLR).^7^ Binding triggers internalization through the clathrin pathway.^11^ Decreasing pH within the endosome triggers depolymerization, thereby exposing hydrophobic residues previously buried in the trimer interface, which in turn catalyze a highly efficient fusion reaction between the viral and endosomal lipid membranes and release of virus into the cytoplasm.^12^^,^^13^

Previous studies investigating how human serum inactivates VSV and VSV-G-pseudotyped viral particles led to the identification and characterization of important interactions between virus particles and complement-fixing antibodies in both immune and nonimmune sera.^14^^,^^15^^,^^16^^,^^17^ Natural IgM antibodies in nonimmune human sera bind to VSV-G and trigger the complement cascade. C3b is deposited on the viral surface, compromising its ability to interact with LDLR and recruiting the C5-9 membrane attack complex, which lyses the viral membrane.^14^^,^^15^^,^^18^ Virus inactivation by this mechanism is gradual and typically not complete until 30 min after serum addition.^16^ Virus inactivation is not manifest in heat-inactivated (HI) human serum because of its dependency on heat-labile complement proteins.^14^^,^^15^^,^^19^ Those previously exposed to VSV through natural infection (e.g., farmers) or therapy with oncolytic VSV also generate high titers of IgG antibodies (neutralizing antibodies), which rapidly and completely neutralize virus infectivity by blocking LDLR-binding sites on VSV-G and powerfully activating the complement cascade.^20^^,^^21^

The assay format used in previous studies of virus inactivation involved incubating VSV or VSV-G-pseudotyped LVs with serum in a small reaction volume and then serially diluting the mixture into tissue culture medium for determination of residual virus titer. Although this format is ideal for the detection of serum factors that irreversibly neutralize virus infectivity, it is not informative for factors that mediate reversible inhibition, since the viruses recover their infectivity upon dilution of the inhibitory activity. Indeed, using the traditional assay format, previous studies failed to recognize the significant virus neutralizing activity of HI serum.^14^^,^^15^^,^^19^ Here, we used a reconfigured assay to comprehensively evaluate the effect of fresh and HI human sera on VSV and VSV-G-pseudotyped LVs, when sera were retained at a high concentration throughout the period of target cell infection/transduction, more closely modeling the physiological conditions encountered during *in vivo* vector delivery.

Using this approach, we discovered a previously unrecognized mechanism of virus inhibition by nonimmune complement-deficient human serum, wherein the interaction of VSV-G with LDLR is competitively inhibited by one or more serum constituents. Mechanistic studies identified the serum factor mediating this reversible inhibition as the subset of serum lipoproteins, which bind to LDLR via their integral apolipoprotein B-100 (ApoB-100) core, and thereby block interaction of VSV or VSV-G-pseudotyped LVs with the receptor. These lipoprotein particles are highly abundant in human serum (∼10^14^ particles per mL). As predicted from their proposed mechanism of action, the inhibitory activity of these particles could be easily circumvented by displaying a ligand on the surface of the VSV or LV particles to redirect their attachment and entry via an alternate (non-LDLR) endocytosing cell surface receptor.

## Results

### Infectivity of target cells by VSV is competitively inhibited by a heat-stable serum component

**Figure 1:**
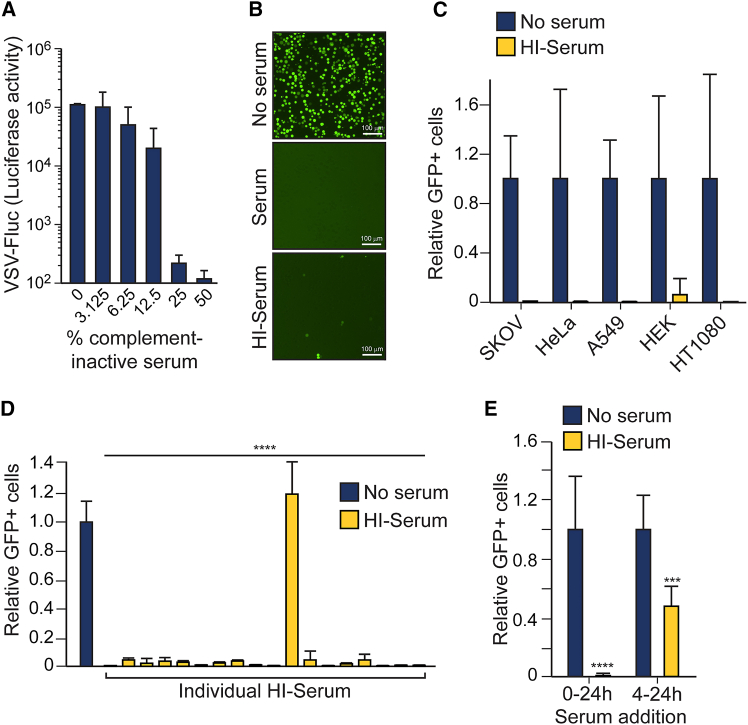
Identification of a heat-stable factor in human serum that competitively inhibits VSV-G-mediated entry (A) Inhibition of VSV by complement-deficient human serum. Vero cells were infected with VSV-Fluc (MOI = 0.01) in the presence of increasing concentrations of naive pooled complement-deficient human serum. After 16 h, luciferase activity was measured. Values represent mean luciferase (with SD) from 2 technical replicates. (B) Heat-inactivated serum inhibits VSV. K562 cells were infected with VSV-GFP (MOI = 0.1) in the presence of medium alone, 25% human serum, or 25% HI human serum. After 24 h, GFP was imaged by fluorescent microscope. (C) Heat-inactivated serum inhibits VSV infectivity on multiple cell lines. The indicated human cell lines were infected with VSV-GFP (MOI = 0.05 for SKOV, HeLa, and HT1080, and 0005 for A549 and HEK) in the presence of medium alone (no serum) or 25% HI human serum. After 24 h, GFP was quantitated. Values represent the number of GFP-positive cells per well (with standard deviation) relative to the medium alone wells for each cell line (*n* = 2 experimental replicates). (D) Analysis of individual human sera. K562 cells were infected with VSV-GFP (MOI = 0.1) in the presence of media alone or 25% HI sera from one of eighteen individual donors. After 24 h, GFP in each well was quantitated. Values represent the number of GFP-positive cells per well (with SD) relative to the medium alone wells (*n* = 2 technical replicates). A *t* test was performed comparing the average median relative GFP value of the individual HI sera to the media alone condition, ∗∗∗∗*p* < 0.0001. (E) Timing of serum addition. K562 cells were infected with VSV-GFP (MOI = 0.1) in the presence of media alone or 25% HI human serum that was added either at the start of inoculation (0–24 h) or 4 h after initial inoculation (4–24 h). Values represent the number of GFP-positive cells per well (with SD) relative to the medium alone wells for each condition (*n* = 8 from two experimental replicates). A one-way ANOVA was performed comparing medium to HI serum for each condition, ∗∗∗*p* < 0.001; ∗∗∗∗*p* < 0.0001.

While complement inactivation of VSV has been extensively studied and is well characterized,^14^^,^^15^ little is known about whether other blood components in nonimmune serum can act as natural barriers to infection. To address this question, we investigated the effect of nonimmune, complement-deficient serum on the transduction of susceptible cells by recombinant VSVs encoding firefly luciferase (Fluc) or enhanced green fluorescent protein (GFP). In the presence of increasing concentrations of nonimmune complement-deficient pooled human serum, VSV-Fluc exhibited a dose-dependent reduction in its ability to infect cultured cells (Figure 1A). Luciferase activity was reduced by ∼500-fold in Vero cells that were infected in the presence of 25% complement-deficient serum compared to Vero cells infected in the absence of serum. Infection of human chronic myelogenous leukemia K562 cells by VSV-GFP was similarly reduced when infected in the presence of 25% HI-pooled human serum (Figure 1B). Virus inhibition by HI serum was confirmed using a larger panel of adherent human tumor cell lines (Figure 1C), with the degree of inhibition ranging from 90% to 99.9% relative to media (no serum) control. We concluded that human serum contains heat-stable virus-inhibitory activity not previously recognized.

Individual HI human sera were tested for virus-inhibitory activity (Figure 1D). Of the 18 sera tested, 17 inhibited >90% of VSV-GFP infectivity, and nearly half of the sera (8/17) inhibited >99% of VSV-GFP infectivity, suggesting the inhibitory activity is a relatively universal component of human serum. Additional experiments indicated that the heat-stable inhibitory activity acted early during infection. When HI serum was added to cells at the same time as virus and left in contact with the cells for 24 h, transduction by VSV-GFP was suppressed to less than 1% of its original titer (Figure 1E). However, when HI serum addition was delayed until 4 h after the cells were exposed to the virus, infection was reduced by only ∼50%, indicating that the HI serum most likely inhibits either attachment or endosomal entry of the virus into target cells.

### Serum lipoproteins (LDL/VLDL) inhibit VSV-G-mediated entry

**Figure 2:**
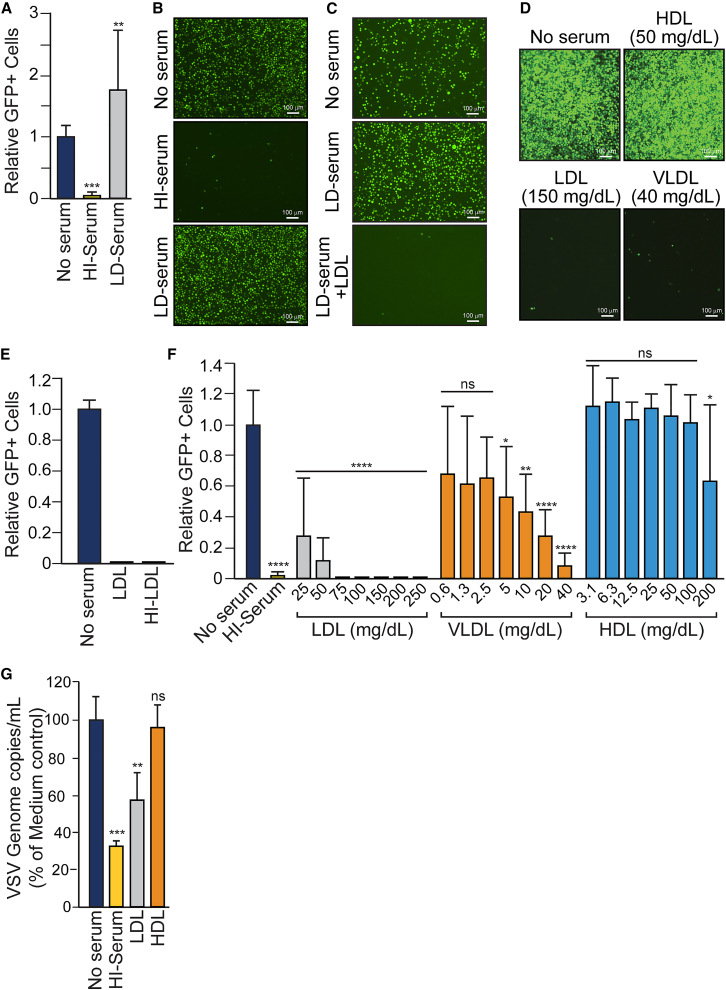
Serum lipoproteins LDL/VLDL competitively inhibit VSV entry (A and B) Lipoprotein-depleted serum does not inhibit VSV. K562 cells were infected with VSV-GFP (MOI = 0.1) in the presence of medium alone, 25% HI human serum, or 25% HI lipoprotein-depleted (LD) serum. After 24 h, GFP was imaged by fluorescent microscope and quantitated by Imaging Cytometry. Values represent the number of GFP-positive cells (with SD) relative to the medium alone (*n* = 12 from 3 experimental replicates). A one-way ANOVA was performed comparing medium to the serum conditions, ∗∗*p* < 0.01; ∗∗∗*p* < 0.001. (C) LDL restores the inhibitory capacity of lipoprotein-depleted serum. K562 cells were infected with VSV-GFP (MOI = 0.1) in the presence of medium alone or 25% HI/LD serum with or without 100 mg/dL of LDL. After 24 h, GFP was imaged by fluorescent microscope. (D) LDL and VLDL but not HDL inhibit VSV. K562 cells were infected with VSV-GFP (MOI = 0.1) in the presence of medium alone, 50 mg/dL HDL, 150 mg/dL LDL, or 40 mg/dL VLDL. After 24 h, GFP was imaged by fluorescent microscope. (E) Inhibition by LDL is heat-stable. K562 cells were infected with VSV-GFP (MOI = 0.1) in the presence of medium alone, 150 mg/dL LDL, or 150 mg/dL heat-treated (56°C) LDL. After 24 h, GFP-positive cells were quantitated. Values represent the number of GFP-positive cells (with SD) relative to the medium alone (*n* = 2). (F) Dose responses. K562 cells were infected with VSV-GFP (MOI = 0.1) in the presence of medium alone, 25% HI serum, or the indicated concentrations (mg/dL) of LDL, VLDL, or HDL. After 24 h, GFP-positive cells were quantitated. Values represent the number of GFP-positive cells (with SD) relative to the medium alone (*n* = 2 experimental replicates). A one-way ANOVA was used to compare the relative GFP-positive cells from the media only conditions against HI serum and lipoprotein dilutions. ns, not significant, ∗*p* < 0.05, ∗∗*p* < 0.01, ∗∗∗∗*p* < 0.0001. (G) Binding assay. HT1080 cells were incubated with VSV-GFP at 4°C in the presence of medium alone, 25% HI serum, or 100 mg/dL LDL. After 90 min, the cells were washed and total RNA was extracted from the samples for quantitation of VSV genomes using a primer-probe set against the VSV-N protein. Values represent the mean percent of genome copies (with SD) relative to the media only control (*n* = 3 from 2 experimental replicates). A one-way ANOVA was performed comparing medium and the different concentrations of LDL, VLDL, and HDL independently. ns, not significant; ∗∗*p* < 0.01; ∗∗∗*p* < 0.001.

Since ApoB-100-containing serum lipoproteins bind to LDLR and are present in high concentrations in serum (∼10^14^ particles/mL), we hypothesized that they might be responsible for the inhibitory effect of HI serum on VSV infection. We therefore sought to determine whether serum lipoproteins might competitively inhibit VSV-G-mediated entry by masking the VSV-G-binding sites on LDLR. To test this hypothesis, commercially sourced lipoprotein-depleted (LD) human serum was tested to determine whether it could inhibit VSV-GFP infection of K562 cells. In contrast to non-depleted HI serum, the LD serum showed no inhibitory activity (Figures 2A and 2B). When LDL was added back to the LD serum at concentrations that mirror the physiological blood levels of LDL, the reconstituted serum inhibited VSV-GFP infection (Figure 2C). Since ApoB-100, the lipoprotein constituent that binds to LDLR, is present on both LDL and VLDL particles (also on intermediate-density [IDL]), but not on HDL, we compared the VSV-inhibitory activities of these serum components at physiologically relevant concentrations. LDL and VLDL potently inhibited VSV-GFP infection, when tested at borderline-high physiological levels of 150 mg/dL and 40 mg/dL, respectively (Figure 2D). In contrast, HDL, which does not bind LDLR, did not inhibit VSV-GFP. The inhibitory activity of LDL was not affected by heat inactivation at 56°C for 1 h (Figure 2E), which further supported the conclusion that ApoB-100-containing lipoprotein particles in serum are responsible for the heat-stable inhibitory activity and that inhibition is a consequence of the masking of LDLR. To determine the relationship between the concentration of LDL and VLDL particles and their VSV inhibitory activities, we conducted dose-response studies in which K562 cells were infected with VSV-GFP in the presence of increasing concentrations of LDL, VLDL, or HDL. LDL and VLDL particles inhibited infection of K562 cells in a concentration-dependent manner, whereas HDL particles did not (Figure 2F). LDL potently blocked infection even at concentrations well below healthy physiological levels (40–100 mg/dL), whereas VLDL was inhibitory only at high physiological concentrations (normal blood levels of 5–40 mg/dL). HDL failed to block VSV-GFP infection even at levels well above the normal physiological range (35–80 mg/dL). We concluded that serum lipoproteins, VLDL and particularly LDL, are potent competitive inhibitors of VSV-G-mediated entry at physiologically relevant levels.

To further confirm that the inhibitory effects of HI serum and LDL were mediated via the competitive masking of LDL receptors on target cells, we performed a virus-binding assay on LDLR-positive cells (Figure 2G). Adherent HT1080 cells were used for this assay to facilitate wash steps. Virus was incubated with the cells on ice (at 4°C) to inhibit LDL receptor endocytosis. (We recognize that this may have also compromised the ability of VSV-G and/or serum lipoproteins to bind to LDLR.) The binding reactions were allowed to proceed in the presence or absence of HI serum or LDL. Cell-bound virus was then quantified using a quantitative reverse-transcription PCR (RT-qPCR) assay designed to detect full-length VSV genomes. Compared to media-only control, both HI serum and LDL at a concentration of 100 mg/dL significantly reduced the binding of VSV to LDLR-positive target cells.

**Figure 3:**
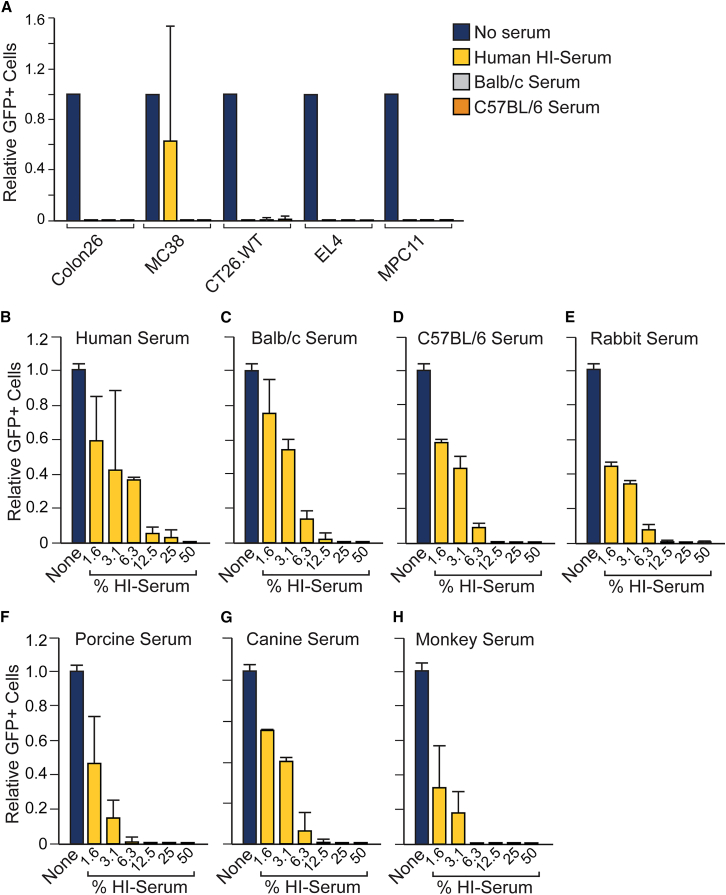
Heat-inactivated animal sera inhibit VSV infection (A) Infection of murine cell lines. A panel of five immortalized murine tumor cell lines were infected with VSV-GFP (MOI = 0.1) in the presence of medium alone, 50% HI human serum, 50% HI Balb/c serum, or 50% HI C57BL/6 serum. After 24 h, GFP-positive cells were quantitated. Values represent the number of GFP-positive cells (with SD) relative to the medium alone for each cell line (*n* = 2 technical replicates). (B–H) Dose response of various animal sera. K562 cell lines were infected with VSV-GFP (MOI = 0.1) in the presence of medium alone or increasing concentrations (percentages) of HI human (B), murine Balb/c (C), murine C57BL/6 (D), rabbit (E), porcine (F), canine (G), or monkey (H) sera. After 24 h, GFP-positive cells were quantitated. Values represent the number of GFP-positive cells (with SD) relative to the medium alone (*n* = from 2 technical replicates).

Since pre-clinical studies of VSV-derived therapies depend on the use of animal models, we evaluated the inhibitory activities of various HI animal sera on the efficiency of VSV infection. Using a panel of five murine cell lines, we initially tested the inhibitory effect of mouse and human HI sera. HI mouse serum, which has lower levels of LDL but higher levels of VLDL than human serum,^22^ potently inhibited VSV-GFP infection in all five cell lines (Figure 3A). HI sera from both Balb/c and C57BL/6 mice were both strongly inhibitory in all murine cell lines, as was HI human serum in four of the five murine cell lines (Figure 3A). Murine 4T1 and B16F10 cells were also tested but not included in the panel as they were poorly infected by VSV-GFP even in the absence of HI serum. We further compared the inhibitory capacity of HI human serum with HI sera obtained from a larger panel of animal species, including mouse, rabbit, pig, dog, and monkey. All of the tested HI sera robustly inhibited infection of K562 cells by VSV-GFP in a dose-dependent manner (Figures 3B–3H). Together, these data demonstrate that competitive inhibition of VSV entry by serum lipoproteins in HI sera is recapitulated in a variety of small and large animal species, including those most commonly used to investigate VSV.

### Engineering the receptor specificity of VSV-G circumvents the inhibitory effect of serum lipoproteins

**Figure 4:**
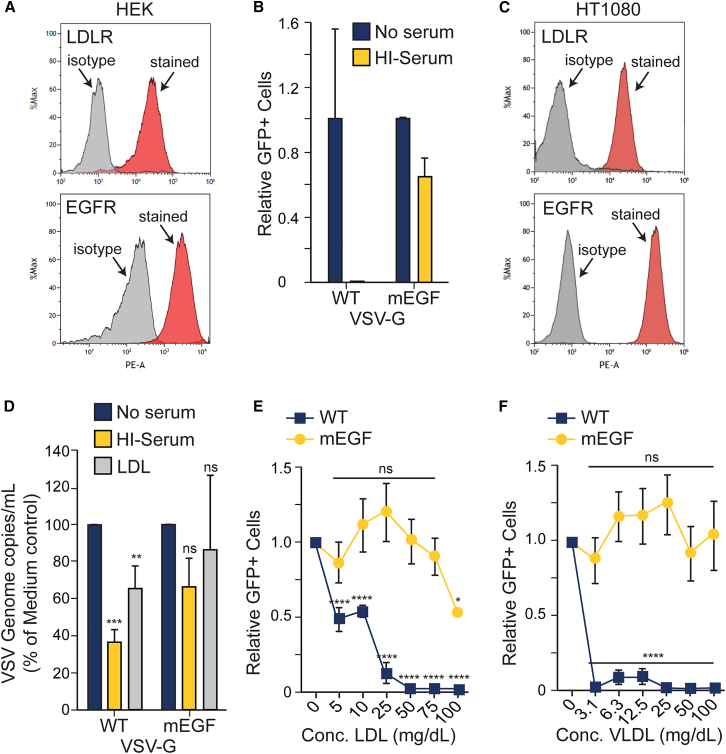
Retargeted VSVs circumvent inhibition by serum lipoproteins (A) Expression of target receptors in HEK-293T cells. HEK-293T cells were stained with isotype control antibody, anti-LDLR antibody (top), or anti-EGFR antibody (bottom) and analyzed by flow cytometry. (B) EGFR-retargeted VSV is resistant to inhibition by HI serum. HEK-293T cells were infected with VSV-GFP containing a wild-type (WT) G (MOI = 0.1) or an EGFR-retargeted G (mEGF; MOI = 1) in the presence of medium alone or 25% HI human serum. After 24 h, GFP-positive cells were quantitated. Values represent number of GFP-positive cells (with SD) relative to the medium alone for each virus (*n* = 2 technical replicates). (C) Expression of target receptors in HT1080 cells. HT1080 cells were stained and analyzed by flow cytometry as described in (A). (D) Binding assay. HT1080 cells were incubated with equal MOIs of VSV-GFP containing WT-G or EGFR-retargeted G at 4°C in the presence of medium alone, 25% HI serum, or 150 mg/dL LDL. After 90 min, the cells were thoroughly washed, and total RNA was extracted from the samples for quantitation of VSV genomes using a primer-probed set against the VSV-N protein. Values represent the mean percent of genome copies (with SD) relative to the media only control (*n* = 6 from two experimental replicates). A one-way ANOVA was performed comparing genome copies in medium to HI serum and LDL conditions for WT and mEGF, ns, not significant; ∗∗*p* < 0.01; ∗∗∗*p* < 0.001. (E and F) EGFR-retargeted VSV is resistant to inhibition by LDL and VLDL. K562-EGFR cells were infected with VSV-GFP containing a WT-G (MOI = 0.1) or an EGFR-retargeted G (MOI = 1) in the presence of increasing concentrations of LDL (E) or VLDL (F). After 24 h, GFP-positive cells were quantitated. Values represent the number of GFP-positive cells (with SD) relative to the medium alone (0 mg/dL) for each virus (*n* = 2 experimental replicates; 2 additional replicates with the same data trend were excluded from the dataset due to differences in potency between lipoprotein lots). A one-way ANOVA was performed independently for each virus curve. The relative GFP^+^ cells were compared at each concentration to the medium only, ns, not significant; ∗*p* < 0.05; ∗∗∗∗*p* < 0.0001.

Since our data indicated that LDL and VLDL can both compete with VSV-G to inhibit its binding to LDLR, which is the primary mediator of virus attachment and entry, we hypothesized that retargeting attachment of the G protein to an alternate cellular receptor might circumvent competitive inhibition by serum lipoproteins. To this end, we compared the infectivity of VSV-GFP containing either a wild-type G (WT-G) glycoprotein or an epidermal growth factor receptor (EGFR)-retargeted G protein in the presence or absence of HI serum. The G protein was “retargeted” to EGFR by displaying a high-affinity variant of EGF as an N-terminal extension of the G protein.^23^^,^^24^ To further compromise its ability to interact with LDLR, two point mutations were introduced into the G protein (K47Q/R354Q).^7^ When used to infect HEK-293 T cells, which naturally express both LDLR and EGFR (Figure 4A), EGFR-retargeted and WT-G viruses both readily infected cells in the absence of serum (Figure 4B). However, when cells were infected in the presence of 25% HI human serum, the WT-G virus was fully inhibited (as expected), while the EGFR-retargeted virus retained full infectivity. In virus-cell-binding assays, attachment of the EGFR-retargeted virus to adherent LDLR and EGFR-expressing HT1080 cells (Figure 4C) was not significantly inhibited by HI human serum nor by purified LDL, which both reduced attachment of the WT-G LDLR-tropic virus (Figure 4D). We next tested the inhibitory effects of increasing concentrations of LDL or VLDL on infection of K562 cells engineered to express EGFR (K562-EGFR; Figure S1). Infection of these cells by parental VSV-GFP was readily inhibited by increasing concentrations of LDL or VLDL, whereas infection by the EGFR-retargeted counterpart of VSV-GFP was unaffected by either lipoprotein (Figures 4E and 4F). These data demonstrate that by retargeting the attachment and entry of VSV through an alternative, non-LDL, receptor can circumvent the competitive inhibitory effect of serum lipoproteins.

**Figure 5:**
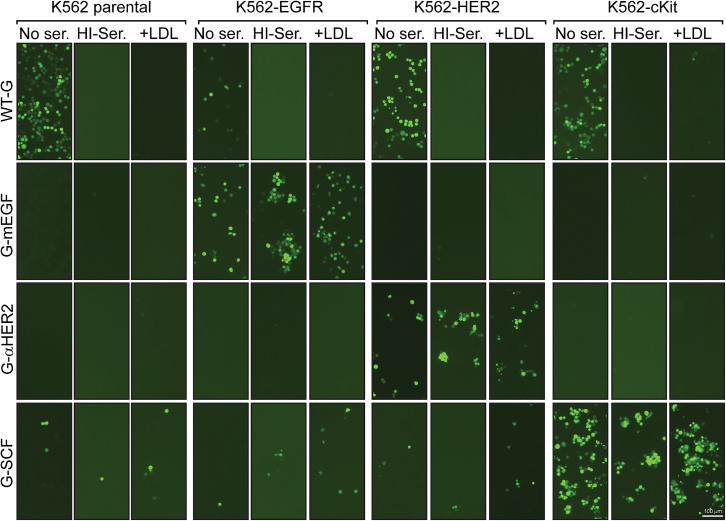
Retargeting to multiple receptors effectively circumvents inhibition by serum lipoproteins K562 parental, K562-EGFR, K562-HER2, or K562-cKit cells were infected with VSV-GFP containing a wild-type (WT) G (MOI = 0.1), an EGFR-retargeted G (G-mEGF; MOI = 1), a HER2-retargeted G (G-αHER2; MOI = 1), or a cKit-retargeted G (G-SCF; MOI = 1). Infections were carried out in the presence of medium alone, 25% HI serum, or 150 mg/dL LDL. After 24 h, cells were imaged using a fluorescent microscope.

To confirm that the inhibitory effect of human serum could be circumvented by retargeting virus entry through cellular receptors other than EGFR, we generated GFP-encoding VSVs in which the G protein was engineered to display an N-terminal α-HER2 scFv or the cKit ligand stem cell factor (SCF) to target entry via the HER2 or cKit stem cell receptor. To facilitate testing of these viruses, a K562 cell panel was generated by lentivirus transduction of K562 cells and selecting for high levels of EGFR, HER2, or cKit receptor expression (Figure S1). Infection of this cell panel by EGFR-, HER2-, and cKit-retargeted VSVs was then compared to infection by the parental VSV in the absence or presence of HI serum or purified LDL (Figure 5). Since infection via targeted (i.e., non-LDLR) receptors is approximately 10-fold less efficient than infection via LDLR, we adjusted the multiplicity of infection (MOIs) of the targeted and untargeted viruses to normalize their infectivity on this cell panel in the absence of serum. The pattern of cell infection observed in these studies illustrated that the inhibitory activity of HI human serum and purified LDL is readily circumvented by redirecting the attachment and entry of VSV through an alternate cellular receptor. Visual examination of the virus-infected cells showed clear specificity of transduction by each retargeted virus and further showed that, in contrast to the parental untargeted virus, receptor-mediated cell entry of the retargeted viruses was unaffected by HI serum or by 150 mg/dL of LDL. Low-level background infection of cKit-negative K562 cells by the cKit-retargeted virus was observed in this study and is consistent with the low level of cKit expression in these cells (Figure S1). However, transduction by the cKit-retargeted virus was substantially increased on the cKit overexpressing cells and was not inhibited by HI serum, nor by LDL. Quantitation of the virus-infected (GFP^+^) cells in this experiment was performed using imaging cytometry, but the readouts were unreliable since they were heavily impacted by the clustering of infected GFP-positive K562 cells. A virus incorporating an SCF-chimeric G protein, in which SCF was displayed at the N terminus of G but in which LDLR-“blinding” mutations were omitted, was also unaffected by HI serum and by LDL at a concentration of 150 mg/dL (Figure S2), indicating that retargeting rather than the blinding point mutations was responsible for escape from lipoprotein competition. In aggregate, these data indicate that viruses retargeted via the display of a cell-targeting ligand may be useful not only for cell-targeted entry but also for overcoming competitive inhibition of cell attachment and entry by serum lipoproteins.

**Figure 6:**
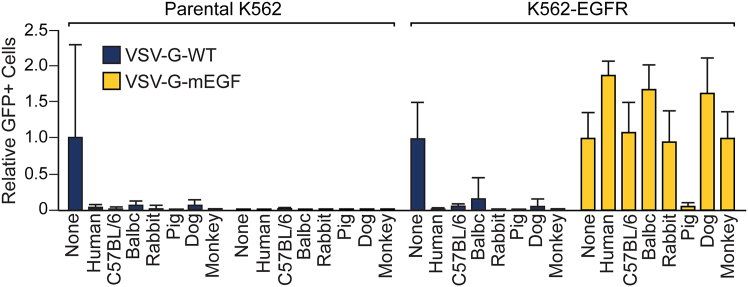
Retargeted VSVs also escape competitive inhibition by serum from other pre-clinical animal models Parental K562 or K562-EGFR cells were infected with VSV-GFP containing a wild-type (WT) G (MOI = 0.1) or an EGFR-retargeted G (mEGF; MOI = 1) in the presence of medium alone or 25% HI serum from the indicated species. After 24 h, GFP-positive cells were quantitated. Values represent the number of GFP-positive cells (with SD) relative to the medium alone (in parental K562 cells for VSV-G-WT and in K562-EGFR cells for VSV-G-mEGF) (*n* = 2 experimental replicates).

To verify that retargeting can circumvent the inhibition of virus attachment and entry by HI sera of non-human origin, we tested HI sera from various animal species to determine whether they could inhibit the EGFR-retargeted VSV. The EGFR-retargeted virus retained its ability to infect EGFR-expressing K562-target cells in the presence of HI sera from human, mouse, rabbit, dog, and monkey (Figure 6). However, pig serum demonstrated potent inhibitory activity against the EGFR-retargeted virus. The mechanism of this inhibition was not further investigated, but it has been well established that, compared to other animal species, pigs have higher concentrations of EGF in their tissues and body fluids,^25^ suggesting a potential for competition between the EGFR-retargeted virus and EGF for EGFR binding.

**Figure 7:**
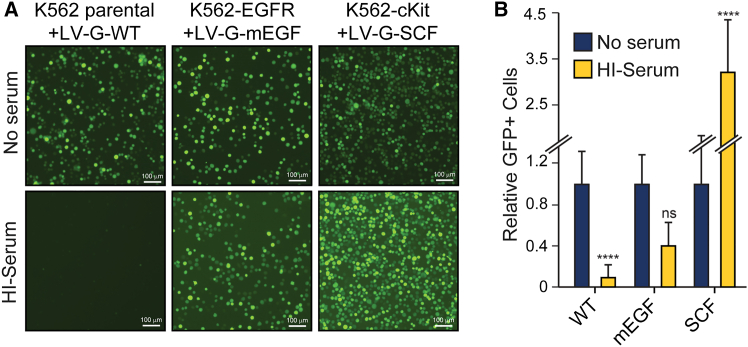
Retargeted VSV-G-pseudotyped lentiviral vectors effectively transduce cells in the presence of serum lipoproteins Parental K562, K562-EGFR, or K562-cKit cells were transduced with LV-GFP containing wild-type (WT) G, EGFR-retargeted G (mEGF), or cKit-retargeted G (SCF), respectively, at 2.8 × 10^4^ lentiviral particles/well, in the presence or absence of 60% heat-inactivated (HI) human serum. After 48 h, GFP-positive cells were imaged using a fluorescence microscope (A) and quantitated by Imaging Cytometry (B). Values represent the number of GFP-positive cells (with SD) relative to the medium alone for each LV (*n* = 4 from 3 experimental replicates). A one-way ANOVA was performed on raw GFP^+^ cell values comparing medium to serum for each LV. ns, not significant; ∗∗∗∗*p* < 0.0001.

Since LVs are now widely used for *ex vivo* gene delivery and editing and have recently attracted attention as a promising platform for direct *in vivo* delivery of CAR transgenes to tissue-resident T lymphocytes, we performed additional studies to determine whether our findings with replication competent VSV would apply equally to VSV-G-pseudotyped LVs. To this end, we generated GFP-encoding LVs pseudotyped with either WT, EGFR-retargeted, or cKit-retargeted G proteins and tested them on an appropriate panel of receptor-positive and -negative cells in the presence or absence of HI human serum. Unsurprisingly, LVs pseudotyped with the WT-G protein were significantly inhibited in the presence of HI human serum (albeit to a lesser degree than replication competent VSVs), but the retargeted LVs were not (Figures 7A and 7B). These data indicate that by retargeting the attachment of the G protein, it is possible to circumvent the serum lipoprotein-mediated inhibition of VSV-G-pseudotyped LVs.

## Discussion

We have shown that LDL and VLDL lipoproteins, both of which are present at high concentrations in human serum, compete with VSV and VSV-G-pseudotyped LV particles for occupancy of cellular LDLR, blocking their LDLR-mediated entry into mammalian cells and reducing their infectious titers 100- to 1,000-fold. Inhibition of virus attachment is immediate after mixing serum with virus and occurs at physiological concentrations of LDL and VLDL. Additionally, the inhibition is circumvented by displaying a targeting molecule fused to the N terminus of the G protein to redirect virus entry through a non-LDL receptor.

Lipoproteins are complex particles with a central core of triglycerides and cholesterol esters surrounded by a shell of phospholipids, free cholesterol, and apolipoproteins. A single copy of ApoB-100, which binds to LDLR, is present on the surface of every VLDL, IDL, and LDL particle.^26^ The Apo B-100-binding site on LDLR maps to CR domains 3, 4, 5, and 6, while the binding site for VSV-G maps to CR domains 2 and 3.^7^^,^^27^^,^^28^ Due to the overlap of these binding sites, it is not surprising that LDL and VLDL particles compete with VSV-G for access to LDLR. Previous studies^14^^,^^15^^,^^17^ may have failed to identify the competitive inhibitory activity of HI serum because they were designed in such a way that they could only detect its irreversible (IgM/complement mediated) inhibitory activity. To expose the competitive, lipoprotein-mediated inhibitory activity of HI serum, it was necessary to expose the target cells to a high concentration of human serum (25% or higher) throughout the time of their exposure to virus.

The “normal” serum concentration of ApoB-100 is 50–150 mg/dL, corresponding to between 1 × 10^14^ and 3 × 10^14^ LDL, IDL, and VLDL particles per milliliter. However, blood concentrations of LDL, IDL, and VLDL vary greatly between individuals. Additionally, within a given individual, blood concentrations of all three lipoproteins can vary significantly over time due to diet, exercise, and cholesterol-lowering drugs, such as statins and PSKC9 inhibitors. Although the serum concentration of VLDL is approximately 10-fold lower than that of LDL (at least in human subjects), VLDL particles also carry multiple copies of the ApoE protein. ApoE binds to CR domain 5 of LDLR^29^ and may therefore enhance avidity and stabilize the binding of VLDL particles to LDLR making it effectively a more potent competitive inhibitor. Interestingly, sera obtained from randomly selected healthy human subjects were highly variable in the efficiencies with which they inhibited VSV-G-mediated infection. We did not observe a strong correlation between VSV-G inhibitory activity and serum levels of LDL or VLDL (Table S1), which is unsurprising given that each serum sample contains a complex mix of LDL, IDL, and VLDL particles, with their concentration profiles being unique to the genetic makeup and metabolic state of the individual donor at the time of the blood draw.

LDL and VLDL were commercially sourced. Since different lots were purified from different human donors, we encountered variability in the potency with which they inhibited VSV infection. The LDL and VLDL preparations purchased for these studies were isolated from human plasma by sequential isopycnic ultracentrifugation using KBr for density adjustments.^30^ While the inhibitory effect of the different lots was consistent, the degree of inhibition varied. This variability can be appreciated by comparing Figure 2F with Figures 4E and 4F, which were conducted using different LDL and VLDL lots. Since LDL and VLDL are part of a continuum that includes IDL particles from which they cannot be accurately separated, we attribute the variability observed between different lipoprotein lots to be a consequence of the natural variance of lipoprotein profiles between the plasma donations from which the particles were “purified.”

We also observed differences in the inhibitory potencies of serum on different target cell lines. We attribute these differences to the variable levels in target cells of both LDLR and LDLR-family members that do not bind to LDL but which are bound by the VSV-G glycoprotein and can mediate VSV entry. Although flow cytometry analysis indicated little variance in LDLR expression levels between the cell lines that we used (Figure S3), we speculate that the lipoproteins present in the added sera may have differentially impacted the kinetics of LDLR internalization and recycling in the different cell lines, thereby amplifying seemingly small variations in surface LDLR expression levels determined in the absence of serum.

Comparison of the inhibitory properties of sera from various nonhuman mammalian species, including mice, indicates that competitive lipoprotein-mediated inhibition of VSV entry is unlikely to be a unique property of human LDL/VLDL. This finding may perhaps help explain why viremic spread of VSV has not been documented in its natural host species (cow, pig, horse, sheep, and goat) and suggests that animal models can be used to recapitulate the inhibitory effect of human serum lipoproteins in pre-clinical studies of *in vivo* vector delivery. Interestingly, although sera from most animal species did not inhibit retargeted viruses entering through a receptor other than LDLR, HI porcine serum blocked VSV with WT-G and EGFR-retargeted G viruses, suggesting an additional inhibitory factor may be present. Since the concentration of EGF in pig tissues and body fluids is known to be elevated,^25^ we hypothesize that this inhibition may be due to the competitive inhibition of virus attachment by serum EGF. In support of this conclusion, antibody neutralization assays did not demonstrate the presence of VSV-neutralizing antibodies in the tested porcine sera.

In pre-clinical mouse studies, the tissue biodistribution of cells infected with intravenously administered VSV or VSV-G-pseudotyped LV particles has been confined predominantly to specific macrophage populations found in the subcapsular lymph-node sinuses and the marginal zone of the spleen.^31^^,^^32^^,^^33^^,^^34^ In light of the data presented in this article, we hypothesize that the restricted biodistribution of systemically administered VSV and VSV-G-pseudotyped vectors in laboratory mice is due to competitive inhibition of LDLR-mediated entry by ApoB-100-containing lipoproteins. The abundance of transduced macrophages observed in the *in vivo* setting suggests that there may be an alternative (non-LDLR-mediated) mechanism whereby VSV and VSV-G-pseudotyped vector particles can enter phagocytic cells.

As described in the introduction, irreversible inhibition of VSV-G-pseudotyped vectors by fresh complement-active human serum is known to be mediated by natural IgM, which binds to VSV-G (Indiana strain) and mediates the neutralization of the viral particles by efficiently recruiting the complement system.^14^^,^^15^^,^^16^^,^^17^ Several approaches have been explored to overcome this mechanism of virus inhibition.^18^^,^^35^^,^^36^^,^^37^^,^^38^^,^^39^^,^^40^^,^^41^^,^^42^^,^^43^ However, since most of the serotypes of VSV and of related vesiculoviruses (e.g., Indiana, New Jersey, Cocal, Carajas, Algoa, and Maraba) are known to enter their target cells through LDLR,^44^^,^^45^ they are all expected to be impacted by the competitive inhibitory effect of serum lipoproteins. We therefore conclude that if VSV or VSV-G-pseudotyped vectors are to be deployed intravenously for direct infection of cancerous tissues or gene delivery and editing applications, their attachment must be retargeted to cellular receptors other than LDLR.

Competitive inhibition of virus entry by serum lipoproteins is immediate, whereas virus inactivation by IgM and complement proceeds more gradually over a period of approximately 30 min.^16^ An IgM/complement-sensitive VSV or VSV-G-pseudotyped vector particle retargeted to a receptor other than LDLR and administered directly into the bloodstream may therefore remain fully infectious for several minutes before it is completely inactivated by IgM and complement. This short window of opportunity may allow retargeted vectors that have been administered into the bloodstream a sufficient survival window to engage and infect target cells before they are completely inactivated by complement.

Regarding the prospect of targeted LVs for systemic *in vivo* gene delivery, our observations were highly encouraging. Vectors displaying an EGF domain at the N terminus of the VSV-G protein and similarly designed vectors displaying SCF or a single-chain antibody against the HER2 receptor (αHER2) demonstrated robust specificity on target-expressing cells. Additionally, HI human serum did not significantly impact their ability to infect EGFR-, cKit-, or HER2 receptor-positive target cells. Our study, therefore, provides additional impetus to the development of fully retargeted LVs for clinical applications focused on direct *in vivo* gene delivery and/or genome editing.

## Materials and methods

### Cells

Cells were maintained at 37°C/5% CO_2_. HEK-293T (a kind gift from Dr. Kah Whye Peng, Mayo Clinic), HeLaH1 (ATCC CRL-1958), A549 (ATCC CCL-185), HT1080 (ATCC CCL-121), CT26.WT (ATCC CRL-2638), B16F10 (ATCC CRL-6475), MC38 (a kind gift from Dr. Stephen Russell, Mayo Clinic), MPC11 (ATCC CCL-167), and EL4 (ATCC TIB-39) cells were maintained in high-glucose DMEM supplemented with 10% fetal bovine serum (FBS) and 100 U penicillin/10 g streptomycin (1× Pen/Strep). Vero (ATCC CCL-81) cells were maintained in high-glucose DMEM supplemented with 5% FBS and 1× Pen/Strep Colon26 (DCTD tumor repository), and 4T1 (ATCC CRL-2539) cells were maintained in RPMI-1640 supplemented with 10% FBS and 1× Pen/Strep. SKOV3ip1 cells (a kind gift from Dr. Kah Whye Peng, Mayo Clinic) were maintained in α-MEM supplemented with 20% FBS and 1× Pen/Strep. All cells acquired from Mayo Clinic were authenticated by short-tandem repeat profiling (STR) analysis, prior to use in experiments. K562 cells (ATCC CCL-243) were maintained in Iscove's Modified Dulbecco's Media (IMDM) supplemented with 15% FBS and 1× Pen/Strep. K562 cells overexpressing EGFR, HER2, or cKit were generated by LV transduction using second generation VSV-G-pseudotyped LVs encoding the receptors under control of the constitutively active spleen focus-forming virus (SFFV) promoter and the puromycin-resistance gene under the phosphoglycerate kinase promoter. Transduced cells were selected using 6 μg/mL puromycin in the media and then clonally selected using ClonaCell TCS (STEMCELL Technologies). Clones were screened by flow cytometry for high expression of the respective receptors.

### Viruses

VSV-GFP and VSV-Fluc with WT-G glycoproteins have been previously described.^46^^,^^47^ VSV-GFP viruses containing G retargeted to EGFR, cKit, or HER2 were rescued on BHK-21 cells as previously described^46^ using newly cloned plasmid DNA VSV genome constructs. To generate the VSV genome constructs, modified VSV-G sequences were synthesized *de novo* by GenScript and used to replace WT-G from the VSV-GFP genome construct using restriction digest. For all constructs, modifications included nucleotide substitutions corresponding to amino-acid mutations K47Q (AAG–CAG) and R354Q (AGG–CAG). For targeting, the sequence for modified EGF,^24^ stem cell factor (GenBank: NP_0008901), or an anti-HER2 scFv^48^ were inserted at the N terminus using no linker (SCF) or a 19 amino acid (AAASGGSGGGGSGGGGSGP) linker (all others). Rescued viruses were amplified to generate stocks, which were titered by TCID50 assay on BHK-21 cells (WT VSV) or Vero cells engineered to overexpress the relevant target receptor and stored at -80°C until use. Briefly, serial 10-fold dilutions of viruses were overlaid onto cell monolayers. After 3–4 days, wells were scored for the presence of CPE and TCID50 titers were calculated using the Spearman-Karber method. Additional construct sequences and information are provided in the supplemental information.

Self-inactivating second-generation LVs were generated by triple transfection of HEK-293T cells with VSV-G envelope plasmid (VSV-G from GenBank: NC_0015601 cloned into a pCG expression vector), second-generation packaging plasmid p8.91,^49^ and eGFP transfer plasmid (Imanis Life Sciences #DNA1023). Briefly, envelope, packaging, and transfer plasmid (in a 1:2:2 ratio) were transfected into HEK-293T monolayers in 15-cm plates using JetPrime transfection reagent (Polyplus). The transfection medium was replaced with serum-free OptiMEM 8–16 h after transfection. Culture supernatants containing the LVs were harvested 72 h after initial transfection and stored at -80°C until use. LV particle titers were determined using a commercially available p24 ELISA reagent system (R&D Systems, Duoset #DY7360-05). Briefly, HIV-1 gag-derived p24 protein concentration was determined from a recombinant p24 protein calibrator using a sandwich ELISA and LV particle number was derived using a factor of 10,000 particles per pg of p24 protein. For targeted LVs, modified VSV-G envelope plasmids were used, where the sequence for modified EGF or SCF were appended to the N terminus of VSV-G, as described for retargeted VSVs. While WT and retargeted LVs had similar p24 titers, functional titers of retargeted LVs were approximately 10-fold lower than those with WT-G. Additional construct sequences and information are provided in the supplemental information.

### Reagents

Pooled human sera were acquired from two sources. For most experiments, pooled human serum off the clot was purchased from Innovative Research (#ISER1000ML). Even prior to heat inactivation, this serum had no detectable complement activity based on CH50 ELISA testing (Microvue CH50 Eq EIA Catalog #A018), likely due to loss of activity during processing. For comparison of complement-active and complement-deficient serum, complement-active pooled human serum was generated in-house using residual research sera collected from individual donors for unrelated studies developing a SARS-CoV-2-neutralizing antibody assay. The clinical protocol to collect blood samples was reviewed and approved by Western IRB on April 1, 2020; study ID: VYR-COV-001. The protocol was conducted under International Conference on Harmonization-Good Clinical Practice (ICH-GCP) and all applicable sections of the Code of Federal Regulations. Samples were obtained with informed consent, and participants agreed that residual samples could be used for other research purposes. Pooled C57BL/6 mouse, Balb/c mouse, canine, monkey, and porcine serum were acquired from Innovative Research. Normal rabbit serum was acquired from Southern Biotech. Lipoprotein-depleted serum was purchased from Sigma-Aldrich (#LP4). To inactivate complement, individual and pooled sera were incubated at 56°C for 60 min. Complement inactivation under these conditions was confirmed in initial tests by CH50 assay (Microvue CH50 Eq EIA Catalog #A018). Human VLDL, HDL, and LDL were purchased from Sigma-Aldrich (#LP1, #LP3-5MG, and #LP2-2MG, respectively). Lipoproteins were used within 1 to 2 months for experiments, as even under appropriate storage conditions, LDL and VLDL exhibited poor stability in solution.

### Serum inhibition assays

Cells were seeded in black-walled clear-bottom 96-well plates. Adherent cells were seeded the day prior to assay, while suspension cells were seeded immediately prior to use in the assays. Cell density at seeding varied between different cell lines to achieve appropriate confluency at the time of assay. For most experiments, K562 cells were used for testing and were seeded at 2 × 10^4^ cells/well. Serum or lipoprotein dilutions were prepared and immediately overlaid onto the cells prior to the addition of virus, unless otherwise noted. After 10 min, virus diluted in serum-free medium was added to the wells. Volumes of cell plating media and virus inoculums were adjusted between experiments to achieve the desired final MOIs and serum/lipoprotein concentrations. MOIs were calculated based on the number of cells at the time of cell seeding and virus titers were calculated by TCID50 assay on BHK-21 or Vero cells. For experiments with retargeted VSVs, a higher MOI was used for the retargeted viruses compared to the WT-G VSV (MOI of 1 versus 01). Because the retargeted viruses displayed lower infectivity on K562 cells relative to the WT virus (Figure S4), using different MOIs was required to achieve similar levels of K562 infection in the presence of media only controls. Unless otherwise noted, assay readout (either luciferase assay or GFP imaging and quantitation) was performed 24 h after the start of infection.

### Microscopy

Phase and GFP images were captured using an Olympus CKX53 microscope and camera at 100× magnification.

### GFP quantitation

Brightfield and GFP fluorescence images of each well were captured using a Celigo Imaging Cytometer (Nexcelom) or Cytation 5 Cell Imaging Multi-Mode Reader (BioTek). Automated identification of GFP-positive cells and quantification of the percent GFP-positive cells were performed using the Celigo or Cytation 5 software. Equivalent analysis and gating parameters were applied across both platforms to ensure consistent quantification.

### Luciferase assay

D-luciferin was added to each well to achieve a final concentration of 3 mg/mL, and luminescence (total RLU [relative light units]) was immediately measured using an Infinite Lumi (Tecan) plate reader.

### Flow cytometry

Flow cytometry was used to quantitate surface expression of receptors. Briefly, cells were stained with PE-conjugated anti-human LDLR (Abcam #FAB2148P), PE-conjugated anti-human EGFR antibody (BioLegend #352904), or the manufacturer recommended isotype control antibodies. After staining, cells were washed, resuspended in buffer consisting of PBS and 2% FBS, and analyzed on a Beckman coulter Cytoflex flow cytometer. The analysis was performed using Kaluza (Beckman-Coulter).

### Binding assays

Dilutions of pooled human serum and virus were overlaid onto HT1080 cells seeded at a density of 1.3 × 10^5^ cells/well in 24-well plates the day before. Following a 90-min incubation at 4°C, the wells were washed three times with cold serum-free medium to remove any non-bound viral particles. Cells were collected by scraping and RNA was extracted using a Universal RNA mini kit (QIAGEN). The amount of viral RNA genomes present in the samples was quantitated by RT-qPCR using primers and probes specific for the intergenic region between VSV-N and P.

### Statistical analyses

Statistical analyses were performed using GraphPad Prism v.10.6.1 (San Diego, CA). One-way analysis of variance (ANOVA) was used to compare means among multiple groups, followed by Tukey’s multiple comparisons test for all pairwise comparisons or Dunnett’s multiple comparisons test when comparing each group to a single control. For comparisons between two independent groups with unequal variances, Welch’s unpaired *t* test was used.

## Data and code availability

Vyriad has ownership over the original data analyzed and displayed in this article. Under specific circumstances, original data may be made available from the corresponding author on request.

## Acknowledgments

This research was carried out at 10.13039/100032691Imanis Life Sciences, 10.13039/100023970LLC, under a research contract from Vyriad, Inc. This contract was funded exclusively by Vyriad for lentivirus studies and by Vyriad and Regeneron for VSV studies.

## Author contributions

R.V. and S.J.R. designed and supervised the overall project direction and wrote the manuscript. R.V. and S.J. designed experiments. S.J., R.N., and C.Z. performed experiments and generated the study data. C.Z. and G.N.N. designed the VSV-G retargeting constructs. S.J., C.Z., G.N.N., R.N., and L.S. generated the retargeted viruses/vectors used in the study. R.V. and S.J. performed the data analysis and generated the figures.

## Declaration of interests

At the time the work was performed, all authors were employees of either Imanis Life Sciences or Vyriad and some (R.V., C.Z., G.N.N., and S.J.R.) are listed as inventors on Vyriad patents. S.J.R. has financial interest in both Imanis Life Sciences and Vyriad. Vyriad has several patents related to retargeting VSV-G glycoprotein for *in vivo* delivery of therapeutics.

## Supplemental information

Document S1. Figures S1–S4 and Tables S1 and S2

Document S2. Article plus supplemental information
