# Supplementary material for: Low-density lipoproteins in human serum competitively inhibit the binding and entry of vesicular stomatitis virus
Source: Mol Ther Adv. 2026 Mar 19;34(2):201721. doi: 10.1016/j.omta.2026.201721 (PMC13148948; doi:10.1016/j.omta.2026.201721)
Supplement: Document S1. Figures S1–S4 and Tables S1 and S2 [file mmc1.pdf]

OMTA, Volume *34*

Supplemental information

Low-density lipoproteins in human serum competitively inhibit the binding and entry of vesicular stomatitis virus

Rianna Vandergaast, Samantha Johnson, Christopher Ziegler, Gopal Naik Nenavath, Luke Schnebeck, Riya Narjari, and Stephen J. Russell

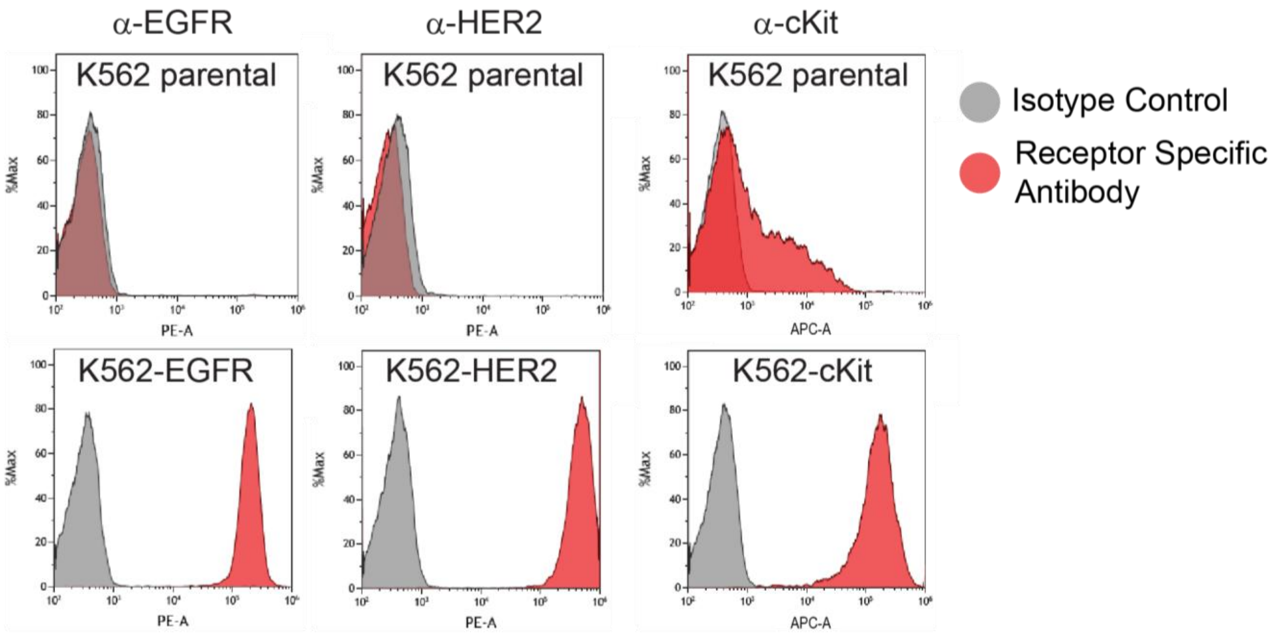

Figure S1: Receptor-specific expression in K562 cells. The indicated K562 cell lines (parental, K562-EGFR, K562-HER2, and K562-cKit) were grown under standard conditions. Cells were collected and stained with PE or APC-conjugated antibodies against human EGFR, HER2, or cKit (BioLegend #324406, 352904, and 313204) or isotype control antibody. Stained cells were subjected to flow cytometry analysis.

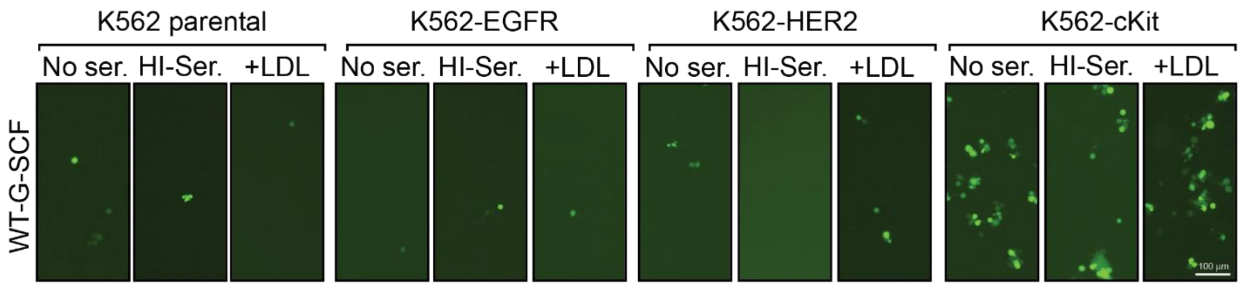

Figure S2: VSV retargeted with SCF but lacking blinding mutations is also resistant to serum inhibition. K562 parental, K562-EGFR, K562-HER2, or K562-cKit cells were infected with VSV-GFP containing a cKit-retargeted G lacking blinding mutations (WT-G-SCF) at an MOI of 1. Infections were carried out in the presence of medium alone, 25% HI serum, or 150 mg/dL LDL. After 24 hours, cells were imaged using a fluorescent microscope. Experiment was performed alongside conditions shown in main Figure 5.

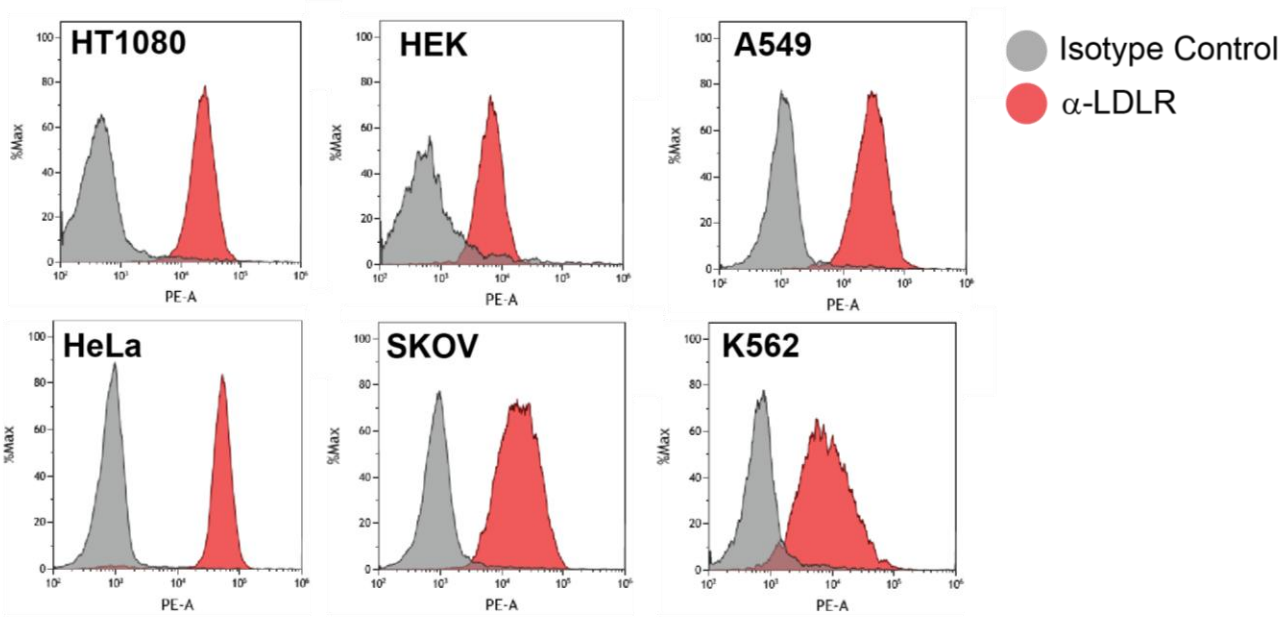

Figure S3: LDLR expression levels in various cell lines. The indicated human cell lines were grown under standard conditions. Cells were collected and stained with PE-conjugated mouse anti-human LDLR antibody (R&D Systems #FAB2148P) or isotype control antibody. Stained cells were subjected to flow cytometry analysis.

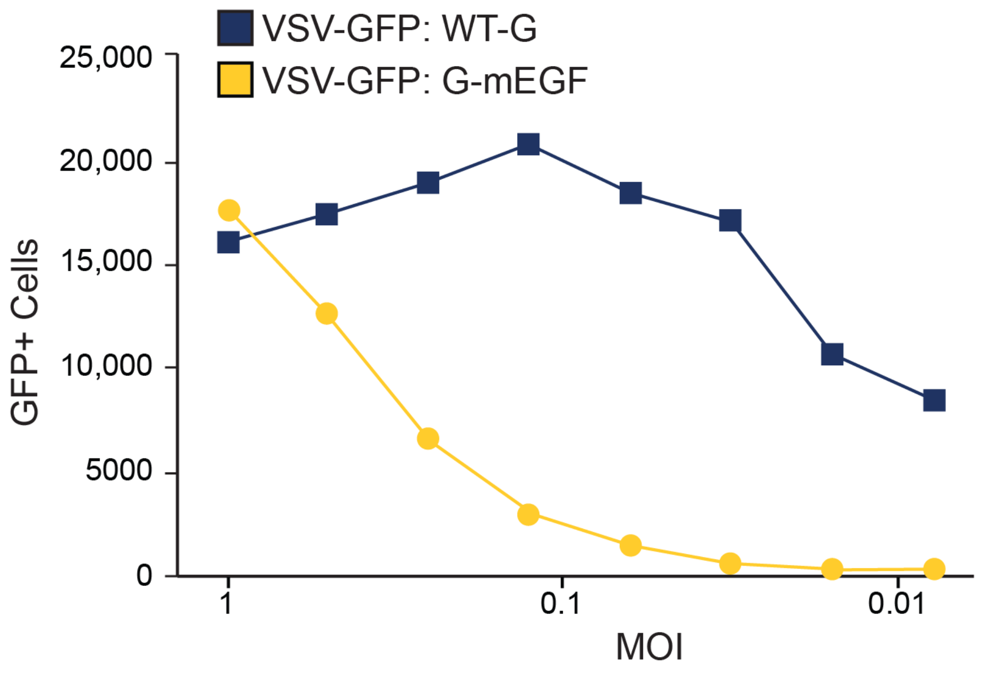

Figure S4: Relative infectivity of VSV-GFP with WT-G and G-mEGF glycoprotein. K562-EGFR cells were infected with two-fold serial dilutions of VSV-GFP: WT-G or VSV-GFP: G-mEGF starting at an MOI of 1, where starting virus preparations for the MOI of 1 condition were determined based on $TCID_{50}$ titers of the viruses on Vero (WT-G) or Vero-EGFR (G-mEGF) cells. After 24 hours, plates were imaged with an imaging cytometer, and the number of GFP+ cells per well determined by the cytometer software. While VSV-GFP: WT-G reached maximum signal (infectivity) at an MOI of 0.1, VSV-GFP: G-mEGF did not reach an equivalent infectivity until infection at MOI of 1.

Table S1: Total cholesterol in individual human sera

Serum	Total cholesterol (mg/dL)*
1	279.8
2	169.7
3	141.6
4	90.4
5	130.7
6	121
7	212.4
8	137
9	78
10	225.2
11	198.5
12	130.8
13	186.4
14	216.9
15	117.7
16	106.5
17	166.3
18	121.8

*Determined using HDL and LDL/VLDL Quantitation Kit (Sigma-Aldrich #MAK045).

Table S2: Additional information for genetic constructs used in study

Number	Construct	Description/Source of Sequence
1	pCG-VSV-G	Wild-type VSV-G sequence (NC_001560.1) was cloned into expression plasmid pCG (Addgene #51476).
2	pCG-mEGF-G-QQ	Construct 1 was modified to introduce K47Q (AAG to CAG) and R354Q (AGG to CAG) mutations into the VSV-G sequence, as well as insert the modified (m123 clone) epidermal growth factor sequence (Lahti, JL *et al.* (2011). FEBS Lett. 585, 1135-1139. https://doi.org/10.1016/j.febslet.2011.03.044) after the VSV-G signal peptide (residue 16).
3	pCG-SCF-G-QQ	Construct 1 was modified to introduce K47Q (AAG to CAG) and R354Q (AGG to CAG) mutations into the VSV-G sequence, as well as insert the stem cell factor sequence (NP_000890.1) and a 19 amino acid linker after the VSV-G signal peptide (residue 16).
4	p8.91	Second generation lentiviral packaging plasmid (Zufferey, R, *et al.* (1997) Biotechnol. 15, 871-875. https://doi.org/10.1038/nbt0997-871).
5	pLV-SFFV-eGFP	Enhanced green fluorescence protein (eGFP) cDNA (U55762.1) was cloned into second generation, self-inactivating lentiviral transfer plasmid containing an SFFV promoter (Imanis Life Sciences).
6	pLV-SFFV-EGFR-PGK-Puro	Construct 5 was modified by replacing the eGFP sequence with EGFR cDNA sequence (X00588.1) followed by the murine phosphoglycerate kinase promoter (LT727296.1) and the puromycin resistance gene (JX861384.1).
7	pLV-SFFV-HER2-PGK-Puro	Construct 6 was modified by replacing the EGFR sequence with the HER2 cDNA sequence (X03363.1).
8	pLV-SFFV-cKit-PGK-Puro	Construct 6 was modified by replacing the EGFR sequence with the cKit cDNA sequence (NM_001093772.2).
9	pVSV-Fluc	Plasmid encoding recombinant VSV genome with firefly luciferase and wild-type G (Kelly, EJ *et al.* (2010) J. Virol. 84, 1550-1562. https://doi.org/10.1128/JVI.01788-09.).
10	pVSV-GFP	Plasmid encoding recombinant VSV genome with green fluorescent protein and wild-type G (Fernandez, M *et al.* (2002) J. Virol. 76, 895-904. https://doi.org/10.1128/JVI.76.2.895-904.2002).
11	pVSV-GFP: mEGF-G-QQ	Construct 10 was modified to introduce K47Q (AAG to CAG) and R354Q (AGG to CAG) mutations into the VSV-G sequence, as well as insert the modified (m123 clone) epidermal growth factor sequence (Lahti, JL *et al.* (2011). FEBS Lett. 585, 1135-1139. https://doi.org/10.1016/j.febslet.2011.03.044) after the VSV-G signal peptide (residue 16).

Number	Construct	Description/Source of Sequence
12	pVSV-GFP: SCF-G-QQ	Construct 10 was modified to introduce K47Q (AAG to CAG) and R354Q (AGG to CAG) mutations into the VSV-G sequence, as well as insert the stem cell factor sequence (NP_000890.1) and a 19 amino acid linker after the VSV-G signal peptide (residue 16).
13	pVSV-GFP: SCF-G-WT	Construct 10 was modified to insert the stem cell factor sequence (NP_000890.1) and a 19 amino acid linker after the VSV-G signal peptide (residue 16).
14	pVSV-GFP: aHER2-G-QQ	Construct 10 was modified to introduce K47Q (AAG to CAG) and R354Q (AGG to CAG) mutations into the VSV-G sequence, as well as insert the sequence for an anti-HER2 scFV (Schier, R *et al.* (1996) J. Mol. Biol. 263, 551-567. https://doi.org/10.1006/jmbi.1996.0598) and an 18 amino acid linker after the VSV-G signal peptide (residue 16).

Sequences

WT-G sequence:

ATGAAGTGCCTTTTGTACTTAGCCTTTTTATTCATTGGGGTGAATTGCAAGTTCACCATAGTTTTTCC
ACACAACCAAAAAGGAAACTGGAAAAATGTTCCTTCTAATTACCATTATTGCCCGTCAAGCTCAG
ATTTAAATTGGCATAATGACTTAATAGGCACAGCCTTACAAGTCAAAATGCCCAAGAGTCACAAGG
CTATTCAAGCAGACGGTTGGATGTGTCATGCTTCCAAATGGGTCACTACTTGTGATTTCCGCTGGT
ATGGACCGAAGTATATAACACATTCCATCCGATCCTTCACTCCATCTGTAGAACAATGCAAGGAAA
GCATTGAACAAACGAAACAAGGAACTTGGCTGAATCCAGGCTTCCCTCCTCAAAGTTGTGGATAT
GCAACTGTGACGGATGCCGAAGCAGTGATTGTCCAGGTGACTCCTCACCATGTGCTGGTTGATG
AATACACAGGAGAATGGGTTGATTCACAGTTCATCAACGGAAAATGCAGCAATTACATATGCCCC
ACTGTCCATAACTCTACAACCTGGCATTCTGACTATAAGGTCAAAGGGCTATGTGATTCTAACCTC
ATTTCCATGGACATCACCTTCTTCTCAGAGGACGGAGAGCTATCATCCCTGGGAAAGGAGGGCA
CAGGGTTCAGAAGTAACTACTTTGCTTATGAAACTGGAGGCAAGGCCTGCAAAATGCAATACTGC
AAGCATTGGGGAGTCAGACTCCCATCAGGTGTCTGGTTCGAGATGGCTGATAAGGATCTCTTTGC
TGCAGCCAGATTCCCTGAATGCCCAGAAGGGTCAAGTATCTCTGCTCCATCTCAGACCTCAGTG
GATGTAAGTCTAATTCAGGACGTTGAGAGGATCTTGGATTATTCCCTCTGCCAAGAAACCTGGAG
CAAAATCAGAGCGGGTCTTCCAATCTCTCCAGTGGATCTCAGCTATCTTGCTCCTAAAAACCCAG
GAACCGGTCCTGCTTTCACCATAATCAATGGTACCCTAAAATACTTTGAGACCAGATACATCAGA
GTCGATATTGCTGCTCCAATCCTCTCAAGAATGGTCGGAATGATCAGTGGAACTACCACAGAAAG
GGAACTGTGGGATGACTGGGCACCATATGAAGACGTGGAAATTGGACCCAATGGAGTTCTGAG
GACCAGTTCAGGATATAAGTTTCCTTTATACATGATTGGACATGGTATGTTGGACTCCGATCTTCAT
CTTAGCTCAAAGGCTCAGGTGTTCGAACATCCTCACATTCAAGACGCTGCTTCGCAACTTCCTGA
TGATGAGAGTTTATTTTTTGGTGATACTGGGCTATCCAAAAATCCAATCGAGCTTGTAGAAGGTTGG
TTCAGTAGTTGGAAAAGCTCTATTGCCTCTTTTTTCTTTATCATAGGGTTAATCATTGGACTATTCTTG
GTTCTCCGAGTTGGTATCCATCTTTGCATTAAATTAAAGCACACCAAGAAAAGACAGATTTATACAG
ACATAGAGATGAACCGACTTGGAAAGTAA

G-mEGF sequence:

ATGAAGTGCCTTTTGTACTTAGCCTTTTTATTCATTGGGGTGAATTGCAAGAACTCCTATTCAGAGTG
TCCGCCGTCCTACGATGGATATTGCCTCCACGACGGGGTTTGCCGCTACATTGAAGCGCTCGA
CTCCTATGCCTGTAATTGTGTCGTGGGCTACGCTGGTGAACGATGCCAATACAGGGACTTGAGAT
GGTGGGGCCGAAGGAAGTTCACGATCGTTTTTCCACACAACCAAAAAGGAAACTGGAAAAATGT
TCCTTCTAATTACCATTATTGCCCGTCAAGCTCAGATTTAAATTGGCATAATGACTTAATAGGCACA
GCCTTACAAGTCAAAATGCCCCAGAGTCACAAGGCTATTCAAGCAGACGGTTGGATGTGTCATG
CTTCCAAATGGGTCACTACTTGTGATTTCCGCTGGTATGGACCGAAGTATATAACACATTCCATCC
GATCCTTCACTCCATCTGTAGAACAATGCAAGGAAAGCATTGAACAAACGAAACAAGGAACTTG
GCTGAATCCAGGCTTCCCTCCTCAAAGTTGTGGATATGCAACTGTGACGGATGCCGAAGCAGTG

ATTGTCCAGGTGACTCCTCACCATGTGCTGGTTGATGAATACACAGGAGAATGGGTTGATTCACA
GTTCATCAACGGAAAATGCAGCAATTACATATGCCCCACTGTCCATAACTCTACAACCTGGCATT
CTGACTATAAGGTCAAAGGGCTATGTGATTCTAACCTCATTTCCATGGACATCACCTTCTTCTCAGA
GGACGGAGAGCTATCATCCCTGGGAAAGGAGGGCACAGGGTTCAGAAGTAACTACTTTGCTTAT
GAAACTGGAGGCAAGGCCTGCAAAATGCAATACTGCAAGCATTGGGGAGTCAGACTCCCATCA
GGTGTCTGGTTCGAGATGGCTGATAAGGATCTCTTTGCTGCAGCCAGATTCCCTGAATGCCCAG
AAGGGTCAAGTATCTCTGCTCCATCTCAGACCTCAGTGGATGTAAGTCTAATTCAGGACGTTGAGA
GGATCTTGGATTATTCCCTCTGCCAAGAAACCTGGAGCAAAATCAGAGCGGGTCTTCCAATCTCT
CCAGTGGATCTCAGCTATCTTGCTCCTAAAAACCCAGGAACCGGTCCTGCTTTCACCATAATCAA
TGGTACCCTAAAATACTTTGAGACCAGATACATCAGAGTCGATATTGCTGCTCCAATCCTCTCAAG
AATGGTCGGAATGATCTCCGGAACTACCACAGAACAGGAACTGTGGGATGACTGGGCGCCATA
TGAAGACGTGGAAATTGGACCCAATGGAGTTCTGAGGACCAGTTCAGGATATAAGTTTCCTTTATA
CATGATTGGACATGGTATGTTGGACTCCGATCTTCATCTTAGCTCAAAGGCTCAGGTGTTCGAACA
TCCTCACATTCAAGACGCTGCTTCGCAACTTCCTGATGATGAGAGTTTATTTTTTGGTGATACTGGG
CTATCCAAAAATCCAATCGAGCTTGTAGAAGGTTGGTTCAGTAGTTGGAAAAGCTCTATTGCCTCT
TTTTTCTTTATCATAGGGTTAATCATTGGACTATTCTTGGTTCTCCGAGTTGGTATCCATCTTTGCATTA
AATTAAAGCACACCAAGAAAAGACAGATTTATACAGACATAGAGATGAACCGACTTGGAAAGTAA

G-αHER2

ATGAAGTGCCTTTTGTACTTAGCCTTTTTATTCATTGGGGTGAATTGCAAGAACCTGAGCGCCCAG
GTGCAGCTGGTGCAGTCTGGGGCAGAGGTGAAAAAGCCCGGGGAGTCTCTGAAGATCTCCTGT
AAGGGTTCTGGATACAGCTTTACCAGCTACTGGATCGCCTGGGTGCGCCAGATGCCCGGGAAA
GGCCTGGAGTACATGGGGCTCATCTATCCTGGTGACTCTGACACCAAATACAGCCCGTCCTTCC
AAGGCCAGGTCACCATCTCAGTCGACAAGTCCGTCAGCACTGCCTACTTGCAATGGAGCAGTC
TGAAGCCCTCGGACAGCGCCGTGTATTTTTGTGCGAGACATGACGTGGGATATTGCACCGACC
GGACTTGCGCAAAGTGGCCTGAATGGCTGGGCGTATGGGGCCAGGGCACCCTGGTCACCGT
CTCCTCAGGTGGAGGCGGTTCAGGCGGAGGTGGCTCTGGCGGTGGCGGATCGCAGTCTGTGT
TGACGCAGCCGCCCTCAGTGTCTGCGGCCCCAGGACAGAAGGTCACCATCTCCTGCTCTGGA
AGCAGCTCCAACATTGGGAATAATTATGTATCCTGGTATCAGCAGCTCCCAGGAACAGCCCCCA
AACTCCTCATCTATGATCACACCAATCGGCCCGCAGGGGTCCCTGACCGATTCTCTGGCTCCA
AGTCTGGCACCTCAGCCTCCCTGGCCATCAGTGGGTTCCGGTCCGAGGATGAGGCTGATTATT
ACTGTGCCTCCTGGGACTACACCCTCTCGGGCTGGGTGTTCGGCGGAGGGACCAAGGTCACC
GTCCTAGGTGGTGGAGGAGGTTCTGGAGGCGGTGGAAGTGGTGGCGGAGGTAGTGGGCCCAA
GTTCACCATAGTTTTTCCACACAACCAAAAAGGAAACTGGAAAAATGTTCCTTCTAATTACCATTAT
TGCCCGTCAAGCTCAGATTTAAATTGGCATAATGACTTAATAGGCACAGCCTTACAAGTCAAAATG
CCCCAGAGTCACAAGGCTATTCAAGCAGACGGTTGGATGTGTCATGCTTCCAAATGGGTCACTA
CTTGTGATTTCCGCTGGTATGGACCGAAGTATATAACACATTCCATCCGATCCTTCACTCCATCTGT
AGAACAATGCAAGGAAAGCATTGAACAAACGAAACAAGGAACTTGGCTGAATCCAGGCTTCCCT

CCTCAAAGTTGTGGATATGCAACTGTGACGGATGCCGAAGCAGTGATTGTCCAGGTGACTCCTC
ACCATGTGCTGGTTGATGAATACACAGGAGAATGGGTTGATTCACAGTTCATCAACGGAAAATGC
AGCAATTACATATGCCCCACTGTCCATAACTCTACAACCTGGCATTCTGACTATAAGGTCAAAGG
GCTATGTGATTCTAACCTCATTTCCATGGACATCACCTTCTTCTCAGAGGACGGAGAGCTATCATC
CCTGGGAAAGGAGGGCACAGGGTTCAGAAGTAACTACTTTGCTTATGAAACTGGAGGCAAGGC
CTGCAAAATGCAATACTGCAAGCATTGGGGAGTCAGACTCCCATCAGGTGTCTGGTTCGAGATG
GCTGATAAGGATCTCTTTGCTGCAGCCAGATTCCCTGAATGCCCAGAAGGGTCAAGTATCTCTG
CTCCATCTCAGACCTCAGTGGATGTAAGTCTAATTCAGGACGTTGAGAGGATCTTGGATTATTCCC
TCTGCCAAGAAACCTGGAGCAAAATCAGAGCGGGTCTTCCAATCTCTCCAGTGGATCTCAGCTA
TCTTGCTCCTAAAAACCCAGGAACCGGTCCTGCTTTCACCATAATCAATGGTACCCTAAAATACTT
TGAGACCAGATACATCAGAGTCGATATTGCTGCTCCAATCCTCTCAAGAATGGTCGGAATGATCT
CCGGAACTACCACAGAACAGGAACTGTGGGATGACTGGGCGCCATATGAAGACGTGGAAATTG
GACCCAATGGAGTTCTGAGGACCAGTTCAGGATATAAGTTTCCTTTATACATGATTGGACATGGTAT
GTTGGACTCCGATCTTCATCTTAGCTCAAAGGCTCAGGTGTTCGAACATCCTCACATTCAAGACG
CTGCTTCGCAACTTCCTGATGATGAGAGTTTATTTTTTGGTGATACTGGGCTATCCAAAAATCCAAT
CGAGCTTGTAGAAGGTTGGTTCAGTAGTTGGAAAAGCTCTATTGCCTCTTTTTTCTTTATCATAGGG
TTAATCATTGGACTATTCTTGGTTCTCCGAGTTGGTATCCATCTTTGCATTAAATTAAAGCACACCAA
GAAAAGACAGATTTATACAGACATAGAGATGAACCGACTTGGAAAGTAA

G-SCF sequence:

ATGAAGTGCCTTTTGTACTTAGCCTTTTTATTCATTGGGGTGAATTGCAAGGAGGGCATCTGCCGC
AACCGCGTGACCAACAACGTGAAGGACGTGACCAAGCTGGTGGCCAACCTGCCCAAGGACTA
CATGATCACCCTGAAGTACGTGCCCGGCATGGACGTGCTGCCCAGCCACTGCTGGATCAGCG
AGATGGTGGTGCAGCTGTCGGACAGCCTGACCGACCTGCTGGACAAGTTCAGCAACATCAGC
GAGGGCCTGAGCAACTACAGCATCATCGACAAGCTGGTGAACATCGTGGACGACCTGGTGGA
GTGCGTGAAGGAGAACAGCAGCAAGGACCTGAAGAAGAGCTTCAAGAGCCCCGAGCCCCGC
CTGTTCACCCCCGAGGAGTTCTTCCGCATCTTCAACCGCAGCATCGACGCCTTCAAGGACTTC
GTGGTGGCCAGCGAGACCAGCGACTGCGTGGTGAGCAGCACCCTGAGCCCCGAGAAGGAC
AGCCGCGTGAGCGTGACCAAGCCCTTCATGCTGCCCCCCGTGGCCGCCAAGTTCACGATCG
TTTTTCCACACAACCAAAAAGGAAACTGGAAAAATGTTCCTTCTAATTACCATTATTGCCCGTCAA
GCTCAGATTTAAATTGGCATAATGACTTAATAGGCACAGCCTTACAAGTCAAAATGCCCCAGAGT
CACAAGGCTATTCAAGCAGACGGTTGGATGTGTCATGCTTCCAAATGGGTCACTACTTGTGATTTC
CGCTGGTATGGACCGAAGTATATAACACATTCCATCCGATCCTTCACTCCATCTGTAGAACAATGC
AAGGAAAGCATTGAACAAACGAAACAAGGAACTTGGCTGAATCCAGGCTTCCCTCCTCAAAGTT
GTGGATATGCAACTGTGACGGATGCCGAAGCAGTGATTGTCCAGGTGACTCCTCACCATGTGCT
GGTTGATGAATACACAGGAGAATGGGTTGATTCACAGTTCATCAACGGAAAATGCAGCAATTACAT
ATGCCCCACTGTCCATAACTCTACAACCTGGCATTCTGACTATAAGGTCAAAGGGCTATGTGATTC
TAACCTCATTTCCATGGACATCACCTTCTTCTCAGAGGACGGAGAGCTATCATCCCTGGGAAAG

GAGGGCACAGGGTTCAGAAGTAACTACTTTGCTTATGAAACTGGAGGCAAGGCCTGCAAAATGC
AATACTGCAAGCATTGGGGAGTCAGACTCCCATCAGGTGTCTGGTTCGAGATGGCTGATAAGGA
TCTCTTTGCTGCAGCCAGATTCCCTGAATGCCCAGAAGGGTCAAGTATCTCTGCTCCATCTCAGA
CCTCAGTGGATGTAAGTCTAATTCAGGACGTTGAGAGGATCTTGGATTATTCCCTCTGCCAAGAAA
CCTGGAGCAAAATCAGAGCGGGTCTTCCAATCTCTCCAGTGGATCTCAGCTATCTTGCTCCTAAA
AACCCAGGAACCGGTCCTGCTTTCACCATAATCAATGGTACCCTAAAATACTTTGAGACCAGATA
CATCAGAGTCGATATTGCTGCTCCAATCCTCTCAAGAATGGTCGGAATGATCTCCGGAACTACCA
CAGAACAGGAACTGTGGGATGACTGGGCGCCATATGAAGACGTGGAAATTGGACCCAATGGAG
TTCTGAGGACCAGTTCAGGATATAAGTTTCCTTTATACATGATTGGACATGGTATGTTGGACTCCGA
TCTTCATCTTAGCTCAAAGGCTCAGGTGTTCGAACATCCTCACATTCAAGACGCTGCTTCGCAAC
TTCCTGATGATGAGAGTTTATTTTTTGGTGATACTGGGCTATCCAAAAATCCAATCGAGCTTGTAGA
AGGTTGGTTCAGTAGTTGGAAAAGCTCTATTGCCTCTTTTTTCTTTATCATAGGGTTAATCATTGGAC
TATTCTTGGTTCTCCGAGTTGGTATCCATCTTTGCATTAAATTAAAGCACACCAAGAAAAGACAGAT
TTATACAGACATAGAGATGAACCGACTTGGAAAGTAA

WT-G-SCF sequence:

ATGAAGTGCCTTTTGTACTTAGCCTTTTTATTCATTGGGGTGAATTGCAAGGAGGGCATCTGCCGC
AACCGCGTGACCAACAACGTGAAGGACGTGACCAAGCTGGTGGCCAACCTGCCCAAGGACTA
CATGATCACCCTGAAGTACGTGCCCGGCATGGACGTGCTGCCCAGCCACTGCTGGATCAGCG
AGATGGTGGTGCAGCTGTCGGACAGCCTGACCGACCTGCTGGACAAGTTCAGCAACATCAGC
GAGGGCCTGAGCAACTACAGCATCATCGACAAGCTGGTGAACATCGTGGACGACCTGGTGGA
GTGCGTGAAGGAGAACAGCAGCAAGGACCTGAAGAAGAGCTTCAAGAGCCCCGAGCCCCGC
CTGTTCACCCCCGAGGAGTTCTTCCGCATCTTCAACCGCAGCATCGACGCCTTCAAGGACTTC
GTGGTGGCCAGCGAGACCAGCGACTGCGTGGTGAGCAGCACCCTGAGCCCCGAGAAGGAC
AGCCGCGTGAGCGTGACCAAGCCCTTCATGCTGCCCCCCGTGGCCGCCGCGGCCGCAAGT
GGTGGTTCTGGAGGCGGTGGAAGTGGTGGCGGAGGTAGTGGGCCCAAGTTCACCATAGTTTTT
CCACACAACCAAAAAGGAAACTGGAAAAATGTTCCTTCTAATTACCATTATTGCCCGTCAAGCTC
AGATTTAAATTGGCATAATGACTTAATAGGCACAGCCTTACAAGTCAAAATGCCCAAGAGTCACAA
GGCTATTCAAGCAGACGGTTGGATGTGTCATGCTTCCAAATGGGTCACTACTTGTGATTTCCGCT
GGTATGGACCGAAGTATATAACACATTCCATCCGATCCTTCACTCCATCTGTAGAACAATGCAAG
GAAAGCATTGAACAAACGAAACAAGGAACTTGGCTGAATCCAGGCTTCCCTCCTCAAAGTTGTG
GATATGCAACTGTGACGGATGCCGAAGCAGTGATTGTCCAGGTGACTCCTCACCATGTGCTGGT
TGATGAATACACAGGAGAATGGGTTGATTCACAGTTCATCAACGGAAAATGCAGCAATTACATATG
CCCCACTGTCCATAACTCTACAACCTGGCATTCTGACTATAAGGTCAAAGGGCTATGTGATTCTAA
CCTCATTTCCATGGACATCACCTTCTTCTCAGAGGACGGAGAGCTATCATCCCTGGGAAAGGAG
GGCACAGGGTTCAGAAGTAACTACTTTGCTTATGAAACTGGAGGCAAGGCCTGCAAAATGCAAT
ACTGCAAGCATTGGGGAGTCAGACTCCCATCAGGTGTCTGGTTCGAGATGGCTGATAAGGATCT
CTTTGCTGCAGCCAGATTCCCTGAATGCCCAGAAGGGTCAAGTATCTCTGCTCCATCTCAGACC

TCAGTGGATGTAAGTCTAATTCAGGACGTTGAGAGGATCTTGGATTATTCCCTCTGCCAAGAAACC
TGGAGCAAAATCAGAGCGGGTCTTCCAATCTCTCCAGTGGATCTCAGCTATCTTGCTCCTAAAAA
CCCAGGAACCGGTCCTGCTTTCACCATAATCAATGGTACCCTAAAATACTTTGAGACCAGATACA
TCAGAGTCGATATTGCTGCTCCAATCCTCTCAAGAATGGTCGGAATGATCAGTGGAACTACCACA
GAAAGGGAACTGTGGGATGACTGGGCACCATATGAAGACGTGGAAATTGGACCCAATGGAGTT
CTGAGGACCAGTTCAGGATATAAGTTTCCTTTATACATGATTGGACATGGTATGTTGGACTCCGATC
TTCATCTTAGCTCAAAGGCTCAGGTGTTCGAACATCCTCACATTCAAGACGCTGCTTCGCAACTT
CCTGATGATGAGAGTTTATTTTTTGGTGATACTGGGCTATCCAAAAATCCAATCGAGCTTGTAGAAG
GTTGGTTCAGTAGTTGGAAAAGCTCTATTGCCTCTTTTTTCTTTATCATAGGGTTAATCATTGGACTAT
TCTTGGTTCTCCGAGTTGGTATCCATCTTTGCATTAAATTAAAGCACACCAAGAAAAGACAGATTTA
TACAGACATAGAGATGAACCGACTTGGAAAGTAA

Lentiviral EGFR transfer plasmid sequence:

GGAAGGGCTAATTCACTCCCAAAGAAGACAAGATATCCTTGATCTGTGGATCTACCACACACAAG
GCTACTTCCCTGATTAGCAGAACTACACACCAGGGCCAGGGGTCAGATATCCACTGACCTTTGG
ATGGTGCTACAAGCTAGTACCAGTTGAGCCAGATAAGGTAGAAGAGGCCAATAAAGGAGAGAAC
ACCAGCTTGTTACACCCTGTGAGCCTGCATGGGATGGATGACCCGGAGAGAGAAGTGTTAGAGT
GGAGGTTTGACAGCCGCCTAGCATTTCATCACGTGGCCCGAGAGCTGCATCCGGAGTACTTCA
AGAACTGCTGATATCGAGCTTGCTACAAGGGACTTTCCGCTGGGGACTTTCCAGGGAGGCGTG
GCCTGGGCGGGACTGGGGAGTGGCGAGCCCTCAGATCCTGCATATAAGCAGCTGCTTTTTGC
CTGTACTGGGTCTCTCTGGTTAGACCAGATCTGAGCCTGGGAGCTCTCTGGCTAACTAGGGAAC
CCACTGCTTAAGCCTCAATAAAGCTTGCCTTGAGTGCTTCAAGTAGTGTGTGCCCGTCTGTTGTGT
GACTCTGGTAACTAGAGATCCCTCAGACCCTTTTAGTCAGTGTGGAAAATCTCTAGCAGTGGCGC
CCGAACAGGGACTTGAAAGCGAAAGGGAAACCAGAGGAGCTCTCTCGACGCAGGACTCGGC
TTGCTGAAGCGCGCACGGCAAGAGGCGAGGGGCGGCGACTGGTGAGTACGCCAAAAATTTT
GACTAGCGGAGGCTAGAAGGAGAGAGATGGGTGCGAGAGCGTCAGTATTAAGCGGGGGAGAA
TTAGATCGCGATGGGAAAAAATTCGGTTAAGGCCAGGGGGAAAGAAAAAATATAAATTAAAACAT
ATAGTATGGGCAAGCAGGGAGCTAGAACGATTCGCAGTTAATCCTGGCCTGTTAGAAACATCAG
AAGGCTGTAGACAAATACTGGGACAGCTACAACCATCCCTTCAGACAGGATCAGAAGAACTTAG
ATCATTATATAATACAGTAGCAACCCTCTATTGTGTGCATCAAAGGATAGAGATAAAAGACACCAAG
GAAGCTTTAGACAAGATAGAGGAAGAGCAAAACAAAAGTAAGACCACCGCACAGCAAGCGGC
CGGCCGCTGATCTTCAGACCTGGAGGAGGAGATATGAGGGACAATTGGAGAAGTGAATTATATA
AATATAAAGTAGTAAAAATTGAACCATTAGGAGTAGCACCCACCAAGGCAAAGAGAAGAGTGGTG
CAGAGAGAAAAAAGAGCAGTGGGAATAGGAGCTTTGTTCCTTGGGTTCTTGGGAGCAGCAGGA
AGCACTATGGGCGCAGCGTCAATGACGCTGACGGTACAGGCCAGACAATTATTGTCTGGTATAG
TGCAGCAGCAGAACAATTTGCTGAGGGCTATTGAGGCGCAACAGCATCTGTTGCAACTCACAGT
CTGGGGCATCAAGCAGCTCCAGGCAAGAATCCTGGCTGTGGAAAGATACCTAAAGGATCAACA
GCTCCTGGGGATTTGGGGTTGCTCTGGAAAACTCATTTGCACCACTGCTGTGCCTTGGAATGCTA

GTTGGAGTAATAAATCTCTGGAACAGATTTGGAATCACACGACCTGGATGGAGTGGGACAGAGAA
ATTAACAATTACACAAGCTTAATACACTCCTTAATTGAAGAATCGCAAAACCAGCAAGAAAAGAAT
GAACAAGAATTATTGGAATTAGATAAATGGGCAAGTTTGTGGAATTGGTTTAACATAACAAATTGGC
TGTGGTATATAAAATTATTCATAATGATAGTAGGAGGCTTGGTAGGTTTAAGAATAGTTTTTGCTGTAC
TTTCTATAGTGAATAGAGTTAGGCAGGGATATTCACCATTATCGTTTCAGACCCACCTCCCAACCC
CGAGGGGACCCGACAGGCCCGAAGGAATAGAAGAAGAAGGTGGAGAGAGAGACAGAGACAG
ATCCATTCGATTAGTGAACGGATCTCGACGGTATCGCCAAATGGCAGTATTCATCCACAATTTTAA
AAGAAAAGGGGGGATTGGGGGGTACAGTGCAGGGGAAAGAATAGTAGACATAATAGCAACAGA
CATACAAACTAAAGAATTACAAAAACAAATTACAAAAATTCAAAATTTTCGGGTTTATTACAGGGAC
AGCAGAGATCCAGTTTGGATCGATAAGCTTGATATCGAATTCCTGCAGCCCCGATAAAATAAAAG
ATTTTATTTAGTCTCCAGAAAAAGGGGGGAATGAAAGACCCCACCTGTAGGTTTGGCAAGCTAGC
TGCAGTAACGCCATTTTGCAAGGCATGGAAAAATACCAAACCAAGAATAGAGAAGTTCAGATCAA
GGGCGGGTACATGAAAATAGCTAACGTTGGGCCAAACAGGATATCTGCGGTGAGCAGTTTCGG
CCCCGGCCCGGGGCCAAGAACAGATGGTCACCGCAGTTTCGGCCCCGGCCCGAGGCCAA
GAACAGATGGTCCCCAGATATGGCCCAACCCTCAGCAGTTTCTTAAGACCCATCAGATGTTTCC
AGGCTCCCCCAAGGACCTGAAATGACCCTGCGCCTTATTTGAATTAACCAATCAGCCTGCTTCT
CGCTTCTGTTCGCGCGCTTCTGCTTCCCGAGCTCTATAAAAGAGCTCACAACCCCTCACTCGG
CGCGCCAGTCCTCCGACAGACTGAGTCGCCCGGGGGGGATCCATGCGACCCTCCGGGACG
GCCGGGGCAGCGCTCCTGGCGCTGCTGGCTGCGCTCTGCCCGGCGAGTCGGGCTCTGGAG
GAAAAGAAAGTTTGCCAAGGCACGAGTAACAAGCTCACGCAGTTGGGCACTTTTGAAGATCATT
TTCTCAGCCTCCAGAGGATGTTCAATAACTGTGAGGTGGTCCTTGGGAATTTGGAAATTACCTATG
TGCAGAGGAATTATGATCTTTCCTTCTTAAAGACCATCCAGGAGGTGGCTGGTTATGTCCTCATTG
CCCTCAACACAGTGGAGCGAATTCCTTTGGAAAACCTGCAGATCATCAGAGGAAATATGTACTAC
GAAAATTCCTATGCCTTAGCAGTCTTATCTAACTATGATGCAAATAAAACCGGACTGAAGGAGCTG
CCCATGAGAAATTTACAGGAAATCCTGCATGGCGCCGTGCGGTTCAGCAACAACCCTGCCCTG
TGCAACGTGGAGAGCATCCAGTGGCGGGACATAGTCAGCAGTGACTTTCTCAGCAACATGTCG
ATGGACTTCCAGAACCACCTGGGCAGCTGCCAAAAGTGTGATCCAAGCTGTCCCAATGGGAGC
TGCTGGGGTGCAGGAGAGGAGAACTGCCAGAAACTGACCAAAATCATCTGTGCCCAGCAGTG
CTCCGGGCGCTGCCGTGGCAAGTCCCCCAGTGACTGCTGCCACAACCAGTGTGCTGCAGGC
TGCACAGGCCCCCGGGAGAGCGACTGCCTGGTCTGCCGCAAATTCCGAGACGAAGCCACGT
GCAAGGACACCTGCCCCCCACTCATGCTCTACAACCCCACCACGTACCAGATGGATGTGAAC
CCCGAGGGCAAATACAGCTTTGGTGCCACCTGCGTGAAGAAGTGTCCCCGTAATTATGTGGTGA
CAGATCACGGCTCGTGCGTCCGAGCCTGTGGGGCCGACAGCTATGAGATGGAGGAAGACGG
CGTCCGCAAGTGTAAGAAGTGCGAAGGGCCTTGCCGCAAAGTGTGTAACGGAATAGGTATTGGT
GAATTTAAAGACTCACTCTCCATAAATGCTACGAATATTAAACACTTCAAAAACTGCACCTCCATCA
GTGGCGATCTCCACATCCTGCCGGTGGCATTTAGGGGTGACTCCTTCACACATACTCCTCCTCT
GGATCCACAGGAACTGGATATTCTGAAAACCGTAAAGGAAATCACAGGGTTTTTGCTGATTCAGG
CTTGGCCTGAAAACAGGACGGACCTCCATGCCTTTGAGAACCTAGAAATCATACGCGGCAGGA

CCAAGCAACATGGTCAGTTTTCTCTTGCAGTCGTCAGCCTGAACATAACATCCTTGGGATTACGC
TCCCTCAAGGAGATAAGTGATGGAGATGTGATAATTTCAGGAAACAAAAATTTGTGCTATGCAAATA
CAATAAACTGGAAAAAACTGTTTGGGACCTCCGGTCAGAAAACCAAAATTATAAGCAACAGAGGT
GAAAACAGCTGCAAGGCCACAGGCCAGGTCTGCCATGCCTTGTGCTCCCCCGAGGGCTGCT
GGGGCCCGGAGCCCAGGGACTGCGTCTCTTGCCGGAATGTCAGCCGAGGCAGGGAATGCGT
GGACAAGTGCAACCTTCTGGAGGGTGAGCCAAGGGAGTTTGTGGAGAACTCTGAGTGCATACA
GTGCCACCCAGAGTGCCTGCCTCAGGCCATGAACATCACCTGCACAGGACGGGGACCAGAC
AACTGTATCCAGTGTGCCCACTACATTGACGGCCCCCACTGCGTCAAGACCTGCCCGGCAGG
AGTCATGGGAGAAAACAACACCCTGGTCTGGAAGTACGCAGACGCCGGCCATGTGTGCCACC
TGTGCCATCCAAACTGCACCTACGGATGCACTGGGCCAGGTCTTGAAGGCTGTCCAACGAATG
GGCCTAAGATCCCGTCCATCGCCACTGGGATGGTGGGGGCCCTCCTCTTGCTGCTGGTGGTG
GCCCTGGGGATCGGCCTCTTCATGCGAAGGCGCCACATCGTTCGGAAGCGCACGCTGCGGA
GGCTGCTGCAGGAGAGGGAGCTTGTGGAGCCTCTTACACCCAGTGGAGAAGCTCCCAACCAA
GCTCTCTTGAGGATCTTGAAGGAAACTGAATTCAAAAAGATCAAAGTGCTGGGCTCCGGTGCGTT
CGGCACGGTGTATAAGGGACTCTGGATCCCAGAAGGTGAGAAAGTTAAAATTCCCGTCGCTATC
AAGGAATTAAGAGAAGCAACATCTCCGAAAGCCAACAAGGAAATCCTCGATGAAGCCTACGTGA
TGGCCAGCGTGGACAACCCCCACGTGTGCCGCCTGCTGGGCATCTGCCTCACCTCCACCGT
GCAACTCATCACGCAGCTCATGCCCTTCGGCTGCCTCCTGGACTATGTCCGGGAACACAAAGA
CAATATTGGCTCCCAGTACCTGCTCAACTGGTGTGTGCAGATCGCAAAGGGCATGAACTACTTG
GAGGACCGTCGCTTGGTGCACCGCGACCTGGCAGCCAGGAACGTACTGGTGAAAACACCGC
AGCATGTCAAGATCACAGATTTTGGGCTGGCCAAACTGCTGGGTGCGGAAGAGAAAGAATACC
ATGCAGAAGGAGGCAAAGTGCCTATCAAGTGGATGGCATTGGAATCAATTTTACACAGAATCTATA
CCCACCAGAGTGATGTCTGGAGCTACGGGGTGACCGTTTGGGAGTTGATGACCTTTGGATCCAA
GCCATATGACGGAATCCCTGCCAGCGAGATCTCCTCCATCCTGGAGAAAGGAGAACGCCTCC
CTCAGCCACCCATATGTACCATCGATGTCTACATGATCATGGTCAAGTGCTGGATGATAGACGCA
GATAGTCGCCCAAAGTTCCGTGAGTTGATCATCGAATTCTCCAAAATGGCCCGAGACCCCCAG
CGCTACCTTGTCATTCAGGGGGATGAAAGAATGCATTTGCCAAGTCCTACAGACTCCAACTTCTA
CCGTGCCCTGATGGATGAAGAAGACATGGACGACGTGGTGGATGCCGACGAGTACCTCATCCC
ACAGCAGGGCTTCTTCAGCAGCCCCTCCACGTCACGGACTCCCCTCCTGAGCTCTCTGAGTG
CAACCAGCAACAATTCCACCGTGGCTTGCATTGATAGAAATGGGCTGCAAAGCTGTCCCATCAA
GGAAGACAGCTTCTTGCAGCGATACAGCTCAGACCCCACAGGCGCCTTGACTGAGGACAGCA
TAGACGACACCTTCCTCCCAGTGCCTGAATACATAAACCAGTCCGTTCCCAAAAGGCCCGCTG
GCTCTGTGCAGAATCCTGTCTATCACAATCAGCCTCTGAACCCCGCGCCCAGCAGAGACCCAC
ACTACCAGGACCCCCACAGCACTGCAGTGGGCAACCCCGAGTATCTCAACACTGTCCAGCCC
ACCTGTGTCAACAGCACATTCGACAGCCCTGCCCACTGGGCCCAGAAAGGCAGCCACCAAAT
TAGCCTGGACAACCCTGACTACCAGCAGGACTTCTTTCCCAAGGAAGCCAAGCCAAATGGCAT
CTTTAAGGGCTCCACAGCTGAAAATGCAGAATACCTAAGGGTCGCGCCACAAAGCAGTGAATTT
ATTGGAGCATGAGCGGCCGCGACTCTAGAGTCGACCTGCAGGCATGCAAGCTTGATATCAAGC

TTATCGATAATCAACCTCTGGATTACAAAATTTGTGAAAGATTGACTGGTATTCTTAACTATGTTGCTC
CTTTTACGCTATGTGGATACGCTGCTTTAATGCCTTTGTATCATGCTATTGCTTCCCGTATGGCTTTC
ATTTTCTCCTCCTTGTATAAATCCTGGTTGCTGTCTCTTTATGAGGAGTTGTGGCCCGTTGTCAGGC
AACGTGGCGTGGTGTGCACTGTGTTTGCTGACGCAACCCCCACTGGTTGGGGCATTGCCACCA
CCTGTCAGCTCCTTTCCGGGACTTTCGCTTTCCCCCTCCCTATTGCCACGGCGGAACTCATCG
CCGCCTGCCTTGCCCGCTGCTGGACAGGGGCTCGGCTGTTGGGCACTGACAATTCCGTGGTG
TTGTCGGGGAAATCATCGTCCTTTCCTTGGCTGCTCGCCTGTGTTGCCACCTGGATTCTGCGCG
GGACGTCCTTCTGCTACGTCCCTTCGGCCCTCAATCCAGCGGACCTTCCTTCCCATCAATTCTA
CCGGGTAGGGGAGGCGCTTTTCCCAAGGCAGTCTGGAGCATGCGCTTTAGCAGCCCCGCTG
GGCACTTGGCGCTACACAAGTGGCCTCTGGCCTCGCACACATTCCACATCCACCGGTAGGCG
CCAACCGGCTCCGTTCTTTGGTGGCCCCTTCGCGCCACCTTCTACTCCTCCCCTAGTCAGGAA
GTTCCCCCCCGCCCCGCAGCTCGCGTCGTGCAGGACGTGACAAATGGAAGTAGCACGTCTC
ACTAGTCTCGTGCAGATGGACAGCACCGCTGAGCAATGGAAGCGGGTAGGCCTTTGGGGCAG
CGGCCAATAGCAGCTTTGCTCCTTCGCTTTCTGGGCTCAGAGGCTGGGAAGGGGTGGGTCCG
GGGGCGGGCTCAGGGGCGGGCTCAGGGGCGGGGCGGGCGCCCGAAGGTCCTCCGGAGG
CCCGGCATTCTGCACGCTTCAAAAGCGCACGTCTGCCGCGCTGTTCTCCTCTTCCTCATCTCC
GGGCCTTTCGACCTGCAGCCCAAGCTTACCATGACCGAGTACAAGCCCACGGTGCGCCTCG
CCACCCGCGACGACGTCCCCAGGGCCGTACGCACCCTCGCCGCCGCGTTCGCCGACTAC
CCCGCCACGCGCCACACCGTCGATCCGGACCGCCACATCGAGCGGGTCACCGAGCTGCAA
GAACTCTTCCTCACGCGCGTCGGGCTCGACATCGGCAAGGTGTGGGTCGCGGACGACGGCG
CCGCGGTGGCGGTCTGGACCACGCCGGAGAGCGTCGAAGCGGGGGCGGTGTTCGCCGAG
ATCGGCCCGCGCATGGCCGAGTTGAGCGGTTCCCGGCTGGCCGCGCAGCAACAGATGGAA
GGCCTCCTGGCGCCGCACCGGCCCAAGGAGCCCGCGTGGTTCCTGGCCACCGTCGGCGT
CTCGCCCGACCACCAGGGCAAGGGTCTGGGCAGCGCCGTCGTGCTCCCCGGAGTGGAGGC
GGCCGAGCGCGCCGGGGTGCCCGCCTTCCTGGAGACCTCCGCGCCCCGCAACCTCCCCT
TCTACGAGCGGCTCGGCTTCACCGTCACCGCCGACGTCGAGGTGCCCGAAGGACCGCGCA
CCTGGTGCATGACCCGCAAGCCCGGTGCCTGACTCGAGGGAATTAATTCGAGCTCGGTACCTT
TAAGACCAATGACTTACAAGGCAGCTGTAGATCTTAGCCACTTTTTAAAAGAAAAGGGGGGACTG
GAAGGGCTAATTCACTCCCAACGAAGACAAGATCTGCTTTTTGCTTGTACTGGGTCTCTCTGGTTA
GACCAGATCTGAGCCTGGGAGCTCTCTGGCTAACTAGGGAACCCACTGCTTAAGCCTCAATAAA
GCTTGCCTTGAGTGCTTCAAGTAGTGTGTGCCCGTCTGTTGTGTGACTCTGGTAACTAGAGATCC
CTCAGACCCTTTTAGTCAGTGTGGAAAATCTCTAGCAGCATCTAGAATTAATTCCGTGTATTCTATA
GTGTCACCTAAATCGTATGTGTATGATACATAAGGTTATGTATTAATTGTAGCCGCGTTCTAACGACA
ATATGTACAAGCCTAATTGTGTAGCATCTGGCTTACTGAAGCAGACCCTATCATCTCTCTCGTAAAC
TGCCGTCAGAGTCGGTTTGGTTGGACGAACCTTTTGAGTTTCTGGTAACGCCGTCCCGCACCC
GGAAATGGTCAGCGAACCAATCAGCAGGGTCATCGCTAGCCAGATCCTCTACGCCGGACGCA
TCGTGGCCGGCATCACCGGCGCCACAGGTGCGGTTGCTGGCGCCTATATCGCCGACATCAC
CGATGGGGAAGATCGGGCTCGCCACTTCGGGCTCATGAGCGCTTGTTTCGGCGTGGGTATGGT

GGCAGGCCCCGTGGCCGGGGGACTGTTGGGCGCCATCTCCTTGCATGCACCATTCCTTGCG
GCGGCGGTGCTCAACGGCCTCAACCTACTACTGGGCTGCTTCCTAATGCAGGAGTCGCATAAG
GGAGAGCGTCGAATGGTGCACTCTCAGTACAATCTGCTCTGATGCCGCATAGTTAAGCCAGCCC
CGACACCCGCCAACACCCGCTGACGCGCCCTGACGGGCTTGTCTGCTCCCGGCATCCGCTT
ACAGACAAGCTGTGACCGTCTCCGGGAGCTGCATGTGTCAGAGGTTTTCACCGTCATCACCGA
AACGCGCGAGACGAAAGGGCCTCGTGATACGCCTATTTTTATAGGTTAATGTCATGATAATAATGG
TTTCTTAGACGTCAGGTGGCACTTTTCGGGGAAATGTGCGCGGAACCCCTATTTGTTTATTTTTCTA
AATACATTCAAATATGTATCCGCTCATGAGACAATAACCCTGATAAATGCTTCAATAATATTGAAAAA
GGAAGAGTATGAGTATTCAACATTTCCGTGTCGCCCTTATTCCCTTTTTTGCGGCATTTTGCCTTCC
TGTTTTTGCTCACCCAGAAACGCTGGTGAAAGTAAAAGATGCTGAAGATCAGTTGGGTGCACGA
GTGGGTTACATCGAACTGGATCTCAACAGCGGTAAGATCCTTGAGAGTTTTCGCCCCGAAGAAC
GTTTTCCAATGATGAGCACTTTTAAAGTTCTGCTATGTGGCGCGGTATTATCCCGTATTGACGCCG
GGCAAGAGCAACTCGGTCGCCGCATACACTATTCTCAGAATGACTTGGTTGAGTACTCACCAGT
CACAGAAAAGCATCTTACGGATGGCATGACAGTAAGAGAATTATGCAGTGCTGCCATAACCATGA
GTGATAACACTGCGGCCAACTTACTTCTGACAACGATCGGAGGACCGAAGGAGCTAACCGCTT
TTTTGCACAACATGGGGGATCATGTAACTCGCCTTGATCGTTGGGAACCGGAGCTGAATGAAGC
CATACCAAACGACGAGCGTGACACCACGATGCCTGTAGCAATGGCAACAACGTTGCGCAAACT
ATTAACTGGCGAACTACTTACTCTAGCTTCCCGGCAACAATTAATAGACTGGATGGAGGCGGATA
AAGTTGCAGGACCACTTCTGCGCTCGGCCCTTCCGGCTGGCTGGTTTATTGCTGATAAATCTGG
AGCCGGTGAGCGTGGGTCTCGCGGTATCATTGCAGCACTGGGGCCAGATGGTAAGCCCTCCC
GTATCGTAGTTATCTACACGACGGGGAGTCAGGCAACTATGGATGAACGAAATAGACAGATCGCT
GAGATAGGTGCCTCACTGATTAAGCATTGGTAACTGTCAGACCAAGTTTACTCATATATACTTTAGA
TTGATTTAAAACTTCATTTTTAATTTAAAAGGATCTAGGTGAAGATCCTTTTTGATAATCTCATGACCAA
AATCCCTTAACGTGAGTTTTCGTTCCACTGAGCGTCAGACCCCGTAGAAAAGATCAAAGGATCTT
CTTGAGATCCTTTTTTTCTGCGCGTAATCTGCTGCTTGCAAACAAAAAAACCACCGCTACCAGCG
GTGGTTTGTTTGCCGGATCAAGAGCTACCAACTCTTTTTCCGAAGGTAACTGGCTTCAGCAGAGC
GCAGATACCAAATACTGTTCTTCTAGTGTAGCCGTAGTTAGGCCACCACTTCAAGAACTCTGTAGC
ACCGCCTACATACCTCGCTCTGCTAATCCTGTTACCAGTGGCTGCTGCCAGTGGCGATAAGTCG
TGTCTTACCGGGTTGGACTCAAGACGATAGTTACCGGATAAGGCGCAGCGGTCGGGCTGAACG
GGGGGTTCGTGCACACAGCCCAGCTTGGAGCGAACGACCTACACCGAACTGAGATACCTACA
GCGTGAGCTATGAGAAAGCGCCACGCTTCCCGAAGGGAGAAAGGCGGACAGGTATCCGGTAA
GCGGCAGGGTCGGAACAGGAGAGCGCACGAGGGAGCTTCCAGGGGGAAACGCCTGGTATC
TTTATAGTCCTGTCGGGTTTCGCCACCTCTGACTTGAGCGTCGATTTTTGTGATGCTCGTCAGGGG
GGCGGAGCCTATGGAAAAACGCCAGCAACGCGGCCTTTTTACGGTTCCTGGCCTTTTGCTGGC
CTTTTGCTCACATGTTCTTTCCTGCGTTATCCCCTGATTCTGTGGATAACCGTATTACCGCCTTTGA
GTGAGCTGATACCGCTCGCCGCAGCCGAACGACCGAGCGCAGCGAGTCAGTGAGCGAGGAA
GCGGAAGAGCGCCCAATACGCAAACCGCCTCTCCCCGCGCGTTGGCCGATTCATTAATGCAG
CTGTGGAATGTGTGTCAGTTAGGGTGTGGAAAGTCCCCAGGCTCCCCAGCAGGCAGAAGTATG

CAAAGCATGCATCTCAATTAGTCAGCAACCAGGTGTGGAAAGTCCCCAGGCTCCCCAGCAGG
CAGAAGTATGCAAAGCATGCATCTCAATTAGTCAGCAACCATAGTCCCGCCCCTAACTCCGCCC
ATCCCGCCCCTAACTCCGCCCAGTTCCGCCCATTCTCCGCCCCATGGCTGACTAATTTTTTTTAT
TTATGCAGAGGCCGAGGCCGCCTCGGCCTCTGAGCTATTCCAGAAGTAGTGAGGAGGCTTTTTT
GGAGGCCTAGGCTTTTGCAAAAAGCTTGGACACAAGACAGGCTTGCGAGATATGTTTGAGAATA
CCACTTTATCCCGCGTCAGGGAGAGGCAGTGCGTAAAAAGACGCGGACTCATGTGAAATACTG
GTTTTTAGTGCGCCAGATCTCTATAATCTCGCGCAACCTATTTTCCCCTCGAACACTTTTTAAGCC
GTAGATAAACAGGCTGGGACACTTCACATGAGCGAAAAATACATCGTCACCTGGGACATGTTGC
AGATCCATGCACGTAAACTCGCAAGCCGACTGATGCCTTCTGAACAATGGAAAGGCATTATTGC
CGTAAGCCGTGGCGGTCTGTACCGGGTGCGTTACTGGCGCGTGAACTGGGTATTCGTCATGTC
GATACCGTTTGTATTTCCAGCTACGATCACGACAACCAGCGCGAGCTTAAAGTGCTGAAACGCG
CAGAAGGCGATGGCGAAGGCTTCATCGTTATTGATGACCTGGTGGATACCGGTGGTACTGCGGT
TGCGATTCGTGAAATGTATCCAAAAGCGCACTTTGTCACCATCTTCGCAAAACCGGCTGGTCGT
CCGCTGGTTGATGACTATGTTGTTGATATCCCGCAAGATACCTGGATTGAACAGCCGTGGGATAT
GGGCGTCGTATTCGTCCCGCCAATCTCCGGTCGCTAATCTTTTCAACGCCTGGCACTGCCGGG
CGTTGTTCTTTTTAACTTCAGGCGGGTTACAATAGTTTCCAGTAAGTATTCTGGAGGCTGCATCCAT
GACACAGGCAAACCTGAGCGAAACCCTGTTCAAACCCCGCTTTAAACATCCTGAAACCTCGAC
GCTAGTCCGCCGCTTTAATCACGGCGCACAACCGCCTGTGCAGTCGGCCCTTGATGGTAAAAC
CATCCCTCACTGGTATCGCATGATTAACCGTCTGATGTGGATCTGGCGCGGCATTGACCCACGC
GAAATCCTCGACGTCCAGGCACGTATTGTGATGAGCGATGCCGAACGTACCGACGATGATTTAT
ACGATACGGTGATTGGCTACCGTGGCGGCAACTGGATTTATGAGTGGGCCCCGGATCTTTGTGA
AGGAACCTTACTTCTGTGGTGTGACATAATTGGACAAACTACCTACAGAGATTTAAAGCTCTAAGG
TAAATATAAAATTTTTAAGTGTATAATGTGTTAAACTACTGATTCTAATTGTTTGTGTATTTTAGATTCCAA
CCTATGGAACTGATGAATGGGAGCAGTGGTGGAATGCCTTTAATGAGGAAAACCTGTTTTGCTCA
GAAGAAATGCCATCTAGTGATGATGAGGCTACTGCTGACTCTCAACATTCTACTCCTCCAAAAAA
GAAGAGAAAGGTAGAAGACCCCAAGGACTTTCCTTCAGAATTGCTAAGTTTTTTGAGTCATGCTGT
GTTTAGTAATAGAACTCTTGCTTGCTTTGCTATTTACACCACAAAGGAAAAAGCTGCACTGCTATAC
AAGAAAATTATGGAAAAATATTCTGTAACCTTTATAAGTAGGCATAACAGTTATAATCATAACATACTG
TTTTTTCTTACTCCACACAGGCATAGAGTGTCTGCTATTAATAACTATGCTCAAAAATTGTGTACCTTT
AGCTTTTTAATTTGTAAAGGGGTTAATAAGGAATATTTGATGTATAGTGCCTTGACTAGAGATCATAAT
CAGCCATACCACATTTGTAGAGGTTTTACTTGCTTTAAAAAACCTCCCACACCTCCCCCTGAACC
TGAAACATAAAATGAATGCAATTGTTGTTGTTAACTTGTTTATTGCAGCTTATAATGGTTACAAATAAA
GCAATAGCATCACAAATTTCACAAATAAAGCATTTTTTTCACTGCATTCTAGTTGTGGTTTGTCCAAA
CTCATCAATGTATCTTATCATGTCTGGATCAACTGGATAACTCAAGCTAACCAAAATCATCCCAAAC
TTCCCACCCCATACCCTATTACCACTGCCAATTACCTGTGGTTTCATTTACTCTAAACCTGTGATTC
CTCTGAATTATTTTCATTTTAAAGAAATTGTATTTGTTAAATATGTACTACAAACTTAGTAGTT

Lentiviral HER2 transfer plasmid sequence:

GGAAGGGCTAATTCACTCCCAAAGAAGACAAGATATCCTTGATCTGTGGATCTACCACACACAAG
GCTACTTCCCTGATTAGCAGAACTACACACCAGGGCCAGGGGTCAGATATCCACTGACCTTTGG
ATGGTGCTACAAGCTAGTACCAGTTGAGCCAGATAAGGTAGAAGAGGCCAATAAAGGAGAGAAC
ACCAGCTTGTTACACCCTGTGAGCCTGCATGGGATGGATGACCCGGAGAGAGAAGTGTTAGAGT
GGAGGTTTGACAGCCGCCTAGCATTTCATCACGTGGCCCGAGAGCTGCATCCGGAGTACTTCA
AGAACTGCTGATATCGAGCTTGCTACAAGGGACTTTCCGCTGGGGACTTTCCAGGGAGGCGTG
GCCTGGGCGGGACTGGGGAGTGGCGAGCCCTCAGATCCTGCATATAAGCAGCTGCTTTTTGC
CTGTACTGGGTCTCTCTGGTTAGACCAGATCTGAGCCTGGGAGCTCTCTGGCTAACTAGGGAAC
CCACTGCTTAAGCCTCAATAAAGCTTGCCTTGAGTGCTTCAAGTAGTGTGTGCCCGTCTGTTGTGT
GACTCTGGTAACTAGAGATCCCTCAGACCCTTTTAGTCAGTGTGGAAAATCTCTAGCAGTGGCGC
CCGAACAGGGACTTGAAAGCGAAAGGGAAACCAGAGGAGCTCTCTCGACGCAGGACTCGGC
TTGCTGAAGCGCGCACGGCAAGAGGCGAGGGGCGGCGACTGGTGAGTACGCCAAAAATTTT
GACTAGCGGAGGCTAGAAGGAGAGAGATGGGTGCGAGAGCGTCAGTATTAAGCGGGGGAGAA
TTAGATCGCGATGGGAAAAAATTCGGTTAAGGCCAGGGGGAAAGAAAAAATATAAATTAAAACAT
ATAGTATGGGCAAGCAGGGAGCTAGAACGATTCGCAGTTAATCCTGGCCTGTTAGAAACATCAG
AAGGCTGTAGACAAATACTGGGACAGCTACAACCATCCCTTCAGACAGGATCAGAAGAACTTAG
ATCATTATATAATACAGTAGCAACCCTCTATTGTGTGCATCAAAGGATAGAGATAAAAGACACCAAG
GAAGCTTTAGACAAGATAGAGGAAGAGCAAAACAAAAGTAAGACCACCGCACAGCAAGCGGC
CGGCCGCTGATCTTCAGACCTGGAGGAGGAGATATGAGGGACAATTGGAGAAGTGAATTATATA
AATATAAAGTAGTAAAAATTGAACCATTAGGAGTAGCACCCACCAAGGCAAAGAGAAGAGTGGTG
CAGAGAGAAAAAAGAGCAGTGGGAATAGGAGCTTTGTTCCTTGGGTTCTTGGGAGCAGCAGGA
AGCACTATGGGCGCAGCGTCAATGACGCTGACGGTACAGGCCAGACAATTATTGTCTGGTATAG
TGCAGCAGCAGAACAATTTGCTGAGGGCTATTGAGGCGCAACAGCATCTGTTGCAACTCACAGT
CTGGGGCATCAAGCAGCTCCAGGCAAGAATCCTGGCTGTGGAAAGATACCTAAAGGATCAACA
GCTCCTGGGGATTTGGGGTTGCTCTGGAAAACTCATTTGCACCACTGCTGTGCCTTGGAATGCTA
GTTGGAGTAATAAATCTCTGGAACAGATTTGGAATCACACGACCTGGATGGAGTGGGACAGAGAA
ATTAACAATTACACAAGCTTAATACACTCCTTAATTGAAGAATCGCAAAACCAGCAAGAAAAGAAT
GAACAAGAATTATTGGAATTAGATAAATGGGCAAGTTTGTGGAATTGGTTTAACATAACAAATTGGC
TGTGGTATATAAAATTATTCATAATGATAGTAGGAGGCTTGGTAGGTTTAAGAATAGTTTTTGCTGTAC
TTTCTATAGTGAATAGAGTTAGGCAGGGATATTCACCATTATCGTTTCAGACCCACCTCCCAACCC
CGAGGGGACCCGACAGGCCCGAAGGAATAGAAGAAGAAGGTGGAGAGAGAGACAGAGACAG
ATCCATTCGATTAGTGAACGGATCTCGACGGTATCGCCAAATGGCAGTATTCATCCACAATTTTAA
AAGAAAAGGGGGGATTGGGGGGTACAGTGCAGGGGAAAGAATAGTAGACATAATAGCAACAGA
CATACAAACTAAAGAATTACAAAAACAAATTACAAAAATTCAAAATTTTCGGGTTTATTACAGGGAC
AGCAGAGATCCAGTTTGGATCGATAAGCTTGATATCGAATTCCTGCAGCCCCGATAAAATAAAAG
ATTTTATTTAGTCTCCAGAAAAAGGGGGGAATGAAAGACCCCACCTGTAGGTTTGGCAAGCTAGC
TGCAGTAACGCCATTTTGCAAGGCATGGAAAAATACCAAACCAAGAATAGAGAAGTTCAGATCAA
GGGCGGGTACATGAAAATAGCTAACGTTGGGCCAAACAGGATATCTGCGGTGAGCAGTTTCGG

CCCCGGCCCGGGGCCAAGAACAGATGGTCACCGCAGTTTCGGCCCCGGCCCGAGGCCAA
GAACAGATGGTCCCCAGATATGGCCCAACCCTCAGCAGTTTCTTAAGACCCATCAGATGTTTCC
AGGCTCCCCCAAGGACCTGAAATGACCCTGCGCCTTATTTGAATTAACCAATCAGCCTGCTTCT
CGCTTCTGTTCGCGCGCTTCTGCTTCCCGAGCTCTATAAAAGAGCTCACAACCCCTCACTCGG
CGCGCCAGTCCTCCGACAGACTGAGTCGCCCGGGGGGGATCCACCGGTCGCCACCATGGA
GCTGGCGGCCTTGTGCCGCTGGGGGCTCCTCCTCGCCCTCTTGCCCCCCGGAGCCGCGAG
CACCCAAGTGTGCACCGGCACAGACATGAAGCTGCGGCTCCCTGCCAGTCCCGAGACCCAC
CTGGACATGCTCCGCCACCTCTACCAGGGCTGCCAGGTGGTGCAGGGAAACCTGGAACTCAC
CTACCTGCCCACCAATGCCAGCCTGTCCTTCCTGCAGGATATCCAGGAGGTGCAGGGCTACGT
GCTCATCGCTCACAACCAAGTGAGGCAGGTCCCACTGCAGAGGCTGCGGATTGTGCGAGGCA
CCCAGCTCTTTGAGGACAACTATGCCCTGGCCGTGCTAGACAATGGAGACCCGCTGAACAATA
CCACCCCTGTCACAGGGGCCTCCCCAGGAGGCCTGCGGGAGCTGCAGCTTCGAAGCCTCA
CAGAGATCTTGAAAGGAGGGGTCTTGATCCAGCGGAACCCCCAGCTCTGCTACCAGGACACG
ATTTTGTGGAAGGACATCTTCCACAAGAACAACCAGCTGGCTCTCACACTGATAGACACCAACC
GCTCTCGGGCCTGCCACCCCTGTTCTCCGATGTGTAAGGGCTCCCGCTGCTGGGGAGAGAGT
TCTGAGGATTGTCAGAGCCTGACGCGCACTGTCTGTGCCGGTGGCTGTGCCCGCTGCAAGGG
GCCACTGCCCACTGACTGCTGCCATGAGCAGTGTGCTGCCGGCTGCACGGGCCCCAAGCAC
TCTGACTGCCTGGCCTGCCTCCACTTCAACCACAGTGGCATCTGTGAGCTGCACTGCCCAGCC
CTGGTCACCTACAACACAGACACGTTTGAGTCCATGCCCAATCCCGAGGGCCGGTATACATTC
GGCGCCAGCTGTGTGACTGCCTGTCCCTACAACTACCTTTCTACGGACGTGGGCTCCTGCACC
CTCGTCTGCCCCCTGCACAACCAAGAGGTGACAGCAGAGGATGGAACACAGCGGTGTGAGAA
GTGCAGCAAGCCCTGTGCCCGAGTGTGCTATGGTCTGGGCATGGAGCACTTGCGAGAGGTGA
GGGCAGTTACCAGTGCCAATATCCAGGAGTTTGCTGGCTGCAAGAAGATCTTTGGGAGCCTGG
CATTTCTGCCGGAGAGCTTTGATGGGGACCCAGCCTCCAACACTGCCCCGCTCCAGCCAGAG
CAGCTCCAAGTGTTTGAGACTCTGGAAGAGATCACAGGTTACCTATACATCTCAGCATGGCCGG
ACAGCCTGCCTGACCTCAGCGTCTTCCAGAACCTGCAAGTAATCCGGGGACGAATTCTGCACA
ATGGCGCCTACTCGCTGACCCTGCAAGGGCTGGGCATCAGCTGGCTGGGGCTGCGCTCACT
GAGGGAACTGGGCAGTGGACTGGCCCTCATCCACCATAACACCCACCTCTGCTTCGTGCACA
CGGTGCCCTGGGACCAGCTCTTTCGGAACCCGCACCAAGCTCTGCTCCACACTGCCAACCG
GCCAGAGGACGAGTGTGTGGGCGAGGGCCTGGCCTGCCACCAGCTGTGCGCCCGAGGGCA
CTGCTGGGGTCCAGGGCCCACCCAGTGTGTCAACTGCAGCCAGTTCCTTCGGGGCCAGGAG
TGCGTGGAGGAATGCCGAGTACTGCAGGGGCTCCCCAGGGAGTATGTGAATGCCAGGCACTG
TTTGCCGTGCCACCCTGAGTGTCAGCCCCAGAATGGCTCAGTGACCTGTTTTGGACCGGAGGC
TGACCAGTGTGTGGCCTGTGCCCACTATAAGGACCCTCCCTTCTGCGTGGCCCGCTGCCCCA
GCGGTGTGAAACCTGACCTCTCCTACATGCCCATCTGGAAGTTTCCAGATGAGGAGGGCGCAT
GCCAGCCTTGCCCCATCAACTGCACCCACTCCTGTGTGGACCTGGATGACAAGGGCTGCCCC
GCCGAGCAGAGAGCCAGCCCTCTGACGTCCATCATCTCTGCGGTGGTTGGCATTCTGCTGGTC
GTGGTCTTGGGGGTGGTCTTTGGCATCCTCATCAAGCGACGGCAGCAGAAGATCCGGAAGTAC

ACGATGCGGAGACTGCTGCAGGAAACGGAGCTGGTGGAGCCGCTGACACCTAGCGGAGCGA
TGCCCAACCAGGCGCAGATGCGAATCCTGAAAGAGACGGAGCTGAGGAAGGTGAAGGTGCTT
GGATCTGGCGCTTTTGGCACAGTCTACAAGGGCATCTGGATACCTGATGGGGAGAATGTGAAAA
TTCCAGTGGCCATCAAAGTGTTGAGGGAAAACACATCCCCCAAAGCCAACAAAGAAATCTTAGA
CGAAGCATACGTGATGGCTGGTGTGGGCTCCCCATATGTCTCCCGCCTTCTGGGCATCTGCCT
GACATCCACGGTGCAGCTGGTGACACAGCTTATGCCCTATGGCTGCCTCTTAGACCATGTCCG
GGAAAACCGCGGACGCCTGGGCTCCCAGGACCTGCTGAACTGGTGTATGCAGATTGCCAAGG
GGATGAGCTACCTGGAGGATGTGCGGCTCGTACACAGGGACTTGGCCGCTCGGAACGTGCTG
GTCAAGAGTCCCAACCATGTCAAAATTACAGACTTCGGGCTGGCTCGGCTGCTGGACATTGAC
GAGACAGAGTACCATGCAGATGGGGGCAAGGTGCCCATCAAGTGGATGGCGCTGGAGTCCAT
TCTCCGCCGGCGGTTCACCCACCAGAGTGATGTGTGGAGTTATGGTGTGACTGTGTGGGAGCT
GATGACTTTTGGGGCCAAACCTTACGATGGCATCCCAGCCCGGGAGATCCCTGACCTGCTGGA
AAAGGGGGAGCGGCTGCCCCAGCCCCCCATCTGCACCATTGATGTCTACATGATCATGGTCAA
ATGTTGGATGATTGACTCTGAATGTCGGCCAAGATTCCGGGAGTTGGTGTCTGAATTCTCCCGCA
TGGCCAGGGACCCCCAGCGCTTTGTGGTCATCCAGAATGAGGACTTGGGCCCAGCCAGTCC
CTTGGACAGCACCTTCTACCGCTCACTGCTGGAGGACGATGACATGGGGGACCTGGTGGATGC
TGAGGAGTATCTGGTACCCCAGCAGGGCTTCTTCTGTCCAGACCCTGCCCCGGGCGCTGGGG
GCATGGTCCACCACAGGCACCGCAGCTCATCTACCAGGAGTGGCGGTGGGGACCTGACACTA
GGGCTGGAGCCCTCTGAAGAGGAGGCCCCCAGGTCTCCACTGGCACCCTCCGAAGGGGCT
GGCTCCGATGTATTTGATGGTGACCTGGGAATGGGGGCAGCCAAGGGGCTGCAAAGCCTCCC
CACACATGACCCCAGCCCTCTACAGCGGTACAGTGAGGACCCCACAGTACCCCTGCCCTCTG
AGACTGATGGCTACGTTGCCCCCCTGACCTGCAGCCCCCAGCCTGAATATGTGAACCAGCCA
GATGTTCGGCCCCAGCCCCCTTCGCCCCGAGAGGGCCCTCTGCCTGCTGCCCGACCTGCTG
GTGCCACTCTGGAAAGGCCCAAGACTCTCTCCCCAGGGAAGAATGGGGTCGTCAAAGACGTTT
TTGCCTTTGGGGGTGCCGTGGAGAACCCCGAGTACTTGACACCCCAGGGAGGAGCTGCCCCT
CAGCCCCACCCTCCTCCTGCCTTCAGCCCAGCCTTCGACAACCTCTATTACTGGGACCAGGA
CCCACCAGAGCGGGGGGCTCCACCCAGCACCTTCAAAGGGACACCTACGGCAGAGAACCC
AGAGTACCTGGGTCTGGACGTGCCAGTGTGAGCGGCCGCGACTCTAGAGTCGACCTGCAGGC
ATGCAAGCTTGATATCAAGCTTATCGATAATCAACCTCTGGATTACAAAATTTGTGAAAGATTGACT
GGTATTCTTAACTATGTTGCTCCTTTTACGCTATGTGGATACGCTGCTTTAATGCCTTTGTATCATGCT
ATTGCTTCCCGTATGGCTTTCATTTTCTCCTCCTTGTATAAATCCTGGTTGCTGTCTCTTTATGAGGA
GTTGTGGCCCGTTGTCAGGCAACGTGGCGTGGTGTGCACTGTGTTTGCTGACGCAACCCCCAC
TGGTTGGGGCATTGCCACCACCTGTCAGCTCCTTTCCGGGACTTTCGCTTTCCCCCTCCCTATT
GCCACGGCGGAACTCATCGCCGCCTGCCTTGCCCGCTGCTGGACAGGGGCTCGGCTGTTGG
GCACTGACAATTCCGTGGTGTTGTCGGGGAAATCATCGTCCTTTCCTTGGCTGCTCGCCTGTGTT
GCCACCTGGATTCTGCGCGGGACGTCCTTCTGCTACGTCCCTTCGGCCCTCAATCCAGCGGA
CCTTCCTTCCCATCAATTCTACCGGGTAGGGGAGGCGCTTTTCCCAAGGCAGTCTGGAGCATG
CGCTTTAGCAGCCCCGCTGGGCACTTGGCGCTACACAAGTGGCCTCTGGCCTCGCACACATT

CCACATCCACCGGTAGGCGCCAACCGGCTCCGTTCTTTGGTGGCCCCTTCGCGCCACCTTCT
ACTCCTCCCCTAGTCAGGAAGTTCCCCCCCGCCCCGCAGCTCGCGTCGTGCAGGACGTGAC
AAATGGAAGTAGCACGTCTCACTAGTCTCGTGCAGATGGACAGCACCGCTGAGCAATGGAAGC
GGGTAGGCCTTTGGGGCAGCGGCCAATAGCAGCTTTGCTCCTTCGCTTTCTGGGCTCAGAGG
CTGGGAAGGGGTGGGTCCGGGGGTGGGCTCAGGGGCGGGCTCAGGGGCGGGGCGGGCG
CCCGAAGGTCCTCCGGAGGCCCGGCATTCTGCACGCTTCAAAAGCGCACGTCTGCCGCGCT
GTTCTCCTCTTCCTCATCTCCGGGCCTTTCGACCTGCAGCCCAAGCTTACCTTAATTAAATGACC
GAGTACAAGCCCACGGTGCGCCTCGCCACCCGCGACGACGTCCCCAGGGCCGTACGCACC
CTCGCCGCCGCGTTCGCCGACTACCCCGCCACGCGCCACACCGTCGATCCGGACCGCCA
CATCGAGCGGGTCACCGAGCTGCAAGAACTCTTCCTCACGCGCGTCGGGCTCGACATCGGC
AAGGTGTGGGTCGCGGACGACGGCGCCGCGGTGGCGGTCTGGACCACGCCGGAGAGCGT
CGAAGCGGGGGCGGTGTTCGCCGAGATCGGCCCGCGCATGGCCGAGTTGAGCGGTTCCCG
GCTGGCCGCGCAGCAACAGATGGAAGGCCTCCTGGCGCCGCACCGGCCCAAGGAGCCCG
CGTGGTTCCTGGCCACCGTCGGCGTCTCGCCCGACCACCAGGGCAAGGGTCTGGGCAGCG
CCGTCGTGCTCCCCGGAGTGGAGGCGGCCGAGCGCGCCGGGGTGCCCGCCTTCCTGGAG
ACCTCCGCGCCCCGCAACCTCCCCTTCTACGAGCGGCTCGGCTTCACCGTCACCGCCGAC
GTCGAGGTGCCCGAAGGACCGCGCACCTGGTGCATGACCCGCAAGCCCGGTGCCTGACTC
GAGGGAATTAATTCGAGCTCGGTACCTTTAAGACCAATGACTTACAAGGCAGCTGTAGATCTTAG
CCACTTTTTAAAAGAAAAGGGGGGACTGGAAGGGCTAATTCACTCCCAACGAAGACAAGATCTG
CTTTTTGCTTGTACTGGGTCTCTCTGGTTAGACCAGATCTGAGCCTGGGAGCTCTCTGGCTAACTA
GGGAACCCACTGCTTAAGCCTCAATAAAGCTTGCCTTGAGTGCTTCAAGTAGTGTGTGCCCGTCT
GTTGTGTGACTCTGGTAACTAGAGATCCCTCAGACCCTTTTAGTCAGTGTGGAAAATCTCTAGCAG
CATCTAGAATTAATTCCGTGTATTCTATAGTGTCACCTAAATCGTATGTGTATGATACATAAGGTTATGT
ATTAATTGTAGCCGCGTTCTAACGACAATATGTACAAGCCTAATTGTGTAGCATCTGGCTTACTGAA
GCAGACCCTATCATCTCTCTCGTAAACTGCCGTCAGAGTCGGTTTGGTTGGACGAACCTTTTGAG
TTTCTGGTAACGCCGTCCCGCACCCGGAAATGGTCAGCGAACCAATCAGCAGGGTCATCGCTA
GCCAGATCCTCTACGCCGGACGCATCGTGGCCGGCATCACCGGCGCCACAGGTGCGGTTGC
TGGCGCCTATATCGCCGACATCACCGATGGGGAAGATCGGGCTCGCCACTTCGGGCTCATGA
GCGCTTGTTTCGGCGTGGGTATGGTGGCAGGCCCCGTGGCCGGGGGACTGTTGGGCGCCAT
CTCCTTGCATGCACCATTCCTTGCGGCGGCGGTGCTCAACGGCCTCAACCTACTACTGGGCTG
CTTCCTAATGCAGGAGTCGCATAAGGGAGAGCGTCGAATGGTGCACTCTCAGTACAATCTGCTC
TGATGCCGCATAGTTAAGCCAGCCCCGACACCCGCCAACACCCGCTGACGCGCCCTGACGG
GCTTGTCTGCTCCCGGCATCCGCTTACAGACAAGCTGTGACCGTCTCCGGGAGCTGCATGTGT
CAGAGGTTTTCACCGTCATCACCGAAACGCGCGAGACGAAAGGGCCTCGTGATACGCCTATTT
TTATAGGTTAATGTCATGATAATAATGGTTTCTTAGACGTCAGGTGGCACTTTTCGGGGAAATGTGC
GCGGAACCCCTATTTGTTTATTTTTCTAAATACATTCAAATATGTATCCGCTCATGAGACAATAACCC
TGATAAATGCTTCAATAATATTGAAAAAGGAAGAGTATGAGTATTCAACATTTCCGTGTCGCCCTTAT
TCCCTTTTTTGCGGCATTTTGCCTTCCTGTTTTTGCTCACCCAGAAACGCTGGTGAAAGTAAAAGA

TGCTGAAGATCAGTTGGGTGCACGAGTGGGTTACATCGAACTGGATCTCAACAGCGGTAAGATC
CTTGAGAGTTTTCGCCCCGAAGAACGTTTTCCAATGATGAGCACTTTTAAAGTTCTGCTATGTGGC
GCGGTATTATCCCGTATTGACGCCGGGCAAGAGCAACTCGGTCGCCGCATACACTATTCTCAGA
ATGACTTGGTTGAGTACTCACCAGTCACAGAAAAGCATCTTACGGATGGCATGACAGTAAGAGAA
TTATGCAGTGCTGCCATAACCATGAGTGATAACACTGCGGCCAACTTACTTCTGACAACGATCGG
AGGACCGAAGGAGCTAACCGCTTTTTTGCACAACATGGGGGATCATGTAACTCGCCTTGATCGT
TGGGAACCGGAGCTGAATGAAGCCATACCAAACGACGAGCGTGACACCACGATGCCTGTAGC
AATGGCAACAACGTTGCGCAAACTATTAACTGGCGAACTACTTACTCTAGCTTCCCGGCAACAAT
TAATAGACTGGATGGAGGCGGATAAAGTTGCAGGACCACTTCTGCGCTCGGCCCTTCCGGCTG
GCTGGTTTATTGCTGATAAATCTGGAGCCGGTGAGCGTGGGTCTCGCGGTATCATTGCAGCACT
GGGGCCAGATGGTAAGCCCTCCCGTATCGTAGTTATCTACACGACGGGGAGTCAGGCAACTAT
GGATGAACGAAATAGACAGATCGCTGAGATAGGTGCCTCACTGATTAAGCATTGGTAACTGTCAG
ACCAAGTTTACTCATATATACTTTAGATTGATTTAAAACTTCATTTTTAATTTAAAAGGATCTAGGTGAA
GATCCTTTTTGATAATCTCATGACCAAAATCCCTTAACGTGAGTTTTCGTTCCACTGAGCGTCAGA
CCCCGTAGAAAAGATCAAAGGATCTTCTTGAGATCCTTTTTTTCTGCGCGTAATCTGCTGCTTGCA
AACAAAAAAACCACCGCTACCAGCGGTGGTTTGTTTGCCGGATCAAGAGCTACCAACTCTTTTT
CCGAAGGTAACTGGCTTCAGCAGAGCGCAGATACCAAATACTGTTCTTCTAGTGTAGCCGTAGTT
AGGCCACCACTTCAAGAACTCTGTAGCACCGCCTACATACCTCGCTCTGCTAATCCTGTTACCA
GTGGCTGCTGCCAGTGGCGATAAGTCGTGTCTTACCGGGTTGGACTCAAGACGATAGTTACCGG
ATAAGGCGCAGCGGTCGGGCTGAACGGGGGGTTCGTGCACACAGCCCAGCTTGGAGCGAAC
GACCTACACCGAACTGAGATACCTACAGCGTGAGCTATGAGAAAGCGCCACGCTTCCCGAAG
GGAGAAAGGCGGACAGGTATCCGGTAAGCGGCAGGGTCGGAACAGGAGAGCGCACGAGGG
AGCTTCCAGGGGGAAACGCCTGGTATCTTTATAGTCCTGTCGGGTTTCGCCACCTCTGACTTGA
GCGTCGATTTTTGTGATGCTCGTCAGGGGGGCGGAGCCTATGGAAAAACGCCAGCAACGCGG
CCTTTTTACGGTTCCTGGCCTTTTGCTGGCCTTTTGCTCACATGTTCTTTCCTGCGTTATCCCCTGA
TTCTGTGGATAACCGTATTACCGCCTTTGAGTGAGCTGATACCGCTCGCCGCAGCCGAACGAC
CGAGCGCAGCGAGTCAGTGAGCGAGGAAGCGGAAGAGCGCCCAATACGCAAACCGCCTCT
CCCCGCGCGTTGGCCGATTCATTAATGCAGCTGTGGAATGTGTGTCAGTTAGGGTGTGGAAAGT
CCCCAGGCTCCCCAGCAGGCAGAAGTATGCAAAGCATGCATCTCAATTAGTCAGCAACCAGGT
GTGGAAAGTCCCCAGGCTCCCCAGCAGGCAGAAGTATGCAAAGCATGCATCTCAATTAGTCAG
CAACCATAGTCCCGCCCCTAACTCCGCCCATCCCGCCCCTAACTCCGCCCAGTTCCGCCCAT
TCTCCGCCCCATGGCTGACTAATTTTTTTTATTTATGCAGAGGCCGAGGCCGCCTCGGCCTCTG
AGCTATTCCAGAAGTAGTGAGGAGGCTTTTTTGGAGGCCTAGGCTTTTGCAAAAAGCTTGGACAC
AAGACAGGCTTGCGAGATATGTTTGAGAATACCACTTTATCCCGCGTCAGGGAGAGGCAGTGCG
TAAAAAGACGCGGACTCATGTGAAATACTGGTTTTTAGTGCGCCAGATCTCTATAATCTCGCGCAA
CCTATTTTCCCCTCGAACACTTTTTAAGCCGTAGATAAACAGGCTGGGACACTTCACATGAGCGA
AAAATACATCGTCACCTGGGACATGTTGCAGATCCATGCACGTAAACTCGCAAGCCGACTGATG
CCTTCTGAACAATGGAAAGGCATTATTGCCGTAAGCCGTGGCGGTCTGTACCGGGTGCGTTACT

GGCGCGTGAACTGGGTATTCGTCATGTCGATACCGTTTGTATTTCCAGCTACGATCACGACAACC
AGCGCGAGCTTAAAGTGCTGAAACGCGCAGAAGGCGATGGCGAAGGCTTCATCGTTATTGATG
ACCTGGTGGATACCGGTGGTACTGCGGTTGCGATTCGTGAAATGTATCCAAAAGCGCACTTTGTC
ACCATCTTCGCAAAACCGGCTGGTCGTCCGCTGGTTGATGACTATGTTGTTGATATCCCGCAAGA
TACCTGGATTGAACAGCCGTGGGATATGGGCGTCGTATTCGTCCCGCCAATCTCCGGTCGCTAA
TCTTTTCAACGCCTGGCACTGCCGGGCGTTGTTCTTTTTAACTTCAGGCGGGTTACAATAGTTTCC
AGTAAGTATTCTGGAGGCTGCATCCATGACACAGGCAAACCTGAGCGAAACCCTGTTCAAACCC
CGCTTTAAACATCCTGAAACCTCGACGCTAGTCCGCCGCTTTAATCACGGCGCACAACCGCCT
GTGCAGTCGGCCCTTGATGGTAAAACCATCCCTCACTGGTATCGCATGATTAACCGTCTGATGTG
GATCTGGCGCGGCATTGACCCACGCGAAATCCTCGACGTCCAGGCACGTATTGTGATGAGCGA
TGCCGAACGTACCGACGATGATTTATACGATACGGTGATTGGCTACCGTGGCGGCAACTGGATT
TATGAGTGGGCCCCGGATCTTTGTGAAGGAACCTTACTTCTGTGGTGTGACATAATTGGACAAACT
ACCTACAGAGATTTAAAGCTCTAAGGTAAATATAAAATTTTTAAGTGTATAATGTGTTAAACTACTGATT
CTAATTGTTTGTGTATTTTAGATTCCAACCTATGGAACTGATGAATGGGAGCAGTGGTGGAATGCCT
TTAATGAGGAAAACCTGTTTTGCTCAGAAGAAATGCCATCTAGTGATGATGAGGCTACTGCTGACT
CTCAACATTCTACTCCTCCAAAAAAGAAGAGAAAGGTAGAAGACCCCAAGGACTTTCCTTCAGA
ATTGCTAAGTTTTTTGAGTCATGCTGTGTTTAGTAATAGAACTCTTGCTTGCTTTGCTATTTACACCAC
AAAGGAAAAAGCTGCACTGCTATACAAGAAAATTATGGAAAAATATTCTGTAACCTTTATAAGTAGG
CATAACAGTTATAATCATAACATACTGTTTTTTCTTACTCCACACAGGCATAGAGTGTCTGCTATTAAT
AACTATGCTCAAAAATTGTGTACCTTTAGCTTTTTAATTTGTAAAGGGGTTAATAAGGAATATTTGATG
TATAGTGCCTTGACTAGAGATCATAATCAGCCATACCACATTTGTAGAGGTTTTACTTGCTTTAAAAA
ACCTCCCACACCTCCCCCTGAACCTGAAACATAAAATGAATGCAATTGTTGTTGTTAACTTGTTTAT
TGCAGCTTATAATGGTTACAAATAAAGCAATAGCATCACAAATTTCACAAATAAAGCATTTTTTTCAC
TGCATTCTAGTTGTGGTTTGTCCAAACTCATCAATGTATCTTATCATGTCTGGATCAACTGGATAACT
CAAGCTAACCAAAATCATCCCAAACTTCCCACCCCATACCCTATTACCACTGCCAATTACCTGTG
GTTTCATTTACTCTAAACCTGTGATTCCTCTGAATTATTTTCATTTTAAAGAAATTGTATTTGTTAAATAT
GTACTACAAACTTAGTAGTT

Lentiviral cKit transfer plasmid sequence:

GCGACTCCTTCCTTCCCATCAATTCTACCGGGTAGGGGAGGCGCTTTTCCCAAGGCAGTCTGG
AGCATGCGCTTTAGCAGCCCCGCTGGGCACTTGGCGCTACACAAGTGGCCTCTGGCCTCGCA
CACATTCCACATCCACCGGTAGGCGCCAACCGGCTCCGTTCTTTGGTGGCCCCTTCGCGCCA
CCTTCTACTCCTCCCCTAGTCAGGAAGTTCCCCCCCGCCCCGCAGCTCGCGTCGTGCAGGAC
GTGACAAATGGAAGTAGCACGTCTCACTAGTCTCGTGCAGATGGACAGCACCGCTGAGCAATG
GAAGCGGGTAGGCCTTTGGGGCAGCGGCCAATAGCAGCTTTGCTCCTTCGCTTTCTGGGCTCA
GAGGCTGGGAAGGGGTGGGTCCGGGGGCGGGCTCAGGGGCGGGCTCAGGGGCGGGGCG
GGCGCCCGAAGGTCCTCCGGAGGCCCGGCATTCTGCACGCTTCAAAAGCGCACGTCTGCCG
CGCTGTTCTCCTCTTCCTCATCTCCGGGCCTTTCGACCTGCAGCCCAAGCTTACCATGACCGA

GTACAAGCCCACGGTGCGCCTCGCCACCCGCGACGACGTCCCCAGGGCCGTACGCACCCT
CGCCGCCGCGTTCGCCGACTACCCCGCCACGCGCCACACCGTCGATCCGGACCGCCACAT
CGAGCGGGTCACCGAGCTGCAAGAACTCTTCCTCACGCGCGTCGGGCTCGACATCGGCAAG
GTGTGGGTCGCGGACGACGGCGCCGCGGTGGCGGTCTGGACCACGCCGGAGAGCGTCGA
AGCGGGGGCGGTGTTCGCCGAGATCGGCCCGCGCATGGCCGAGTTGAGCGGTTCCCGGCT
GGCCGCGCAGCAACAGATGGAAGGCCTCCTGGCGCCGCACCGGCCCAAGGAGCCCGCGT
GGTTCCTGGCCACCGTCGGCGTCTCGCCCGACCACCAGGGCAAGGGTCTGGGCAGCGCCG
TCGTGCTCCCCGGAGTGGAGGCGGCCGAGCGCGCCGGGGTGCCCGCCTTCCTGGAGACCT
CCGCGCCCCGCAACCTCCCCTTCTACGAGCGGCTCGGCTTCACCGTCACCGCCGACGTCG
AGGTGCCCGAAGGACCGCGCACCTGGTGCATGACCCGCAAGCCCGGTGCCTGACTCGAGG
GAATTAATTCGAGCTCGGTACCTTTAAGACCAATGACTTACAAGGCAGCTGTAGATCTTAGCCACT
TTTTAAAAGAAAAGGGGGGACTGGAAGGGCTAATTCACTCCCAACGAAGACAAGATCTGCTTTTT
GCTTGTACTGGGTCTCTCTGGTTAGACCAGATCTGAGCCTGGGAGCTCTCTGGCTAACTAGGGA
ACCCACTGCTTAAGCCTCAATAAAGCTTGCCTTGAGTGCTTCAAGTAGTGTGTGCCCGTCTGTTG
TGTGACTCTGGTAACTAGAGATCCCTCAGACCCTTTTAGTCAGTGTGGAAAATCTCTAGCAGCATC
TAGAATTAATTCCGTGTATTCTATAGTGTCACCTAAATCGTATGTGTATGATACATAAGGTTATGTATTA
ATTGTAGCCGCGTTCTAACGACAATATGTACAAGCCTAATTGTGTAGCATCTGGCTTACTGAAGCA
GACCCTATCATCTCTCTCGTAAACTGCCGTCAGAGTCGGTTTGGTTGGACGAACCTTCTGAGTTT
CTGGTAACGCCGTCCCGCACCCGGAAATGGTCAGCGAACCAATCAGCAGGGTCATCGCTAGC
CAGATCCTCTACGCCGGACGCATCGTGGCCGGCATCACCGGCGCCACAGGTGCGGTTGCTG
GCGCCTATATCGCCGACATCACCGATGGGGAAGATCGGGCTCGCCACTTCGGGCTCATGAGC
GCTTGTTTCGGCGTGGGTATGGTGGCAGGCCCCGTGGCCGGGGGACTGTTGGGCGCCATCTC
CTTGCATGCACCATTCCTTGCGGCGGCGGTGCTCAACGGCCTCAACCTACTACTGGGCTGCTT
CCTAATGCAGGAGTCGCATAAGGGAGAGCGTCGAATGGTGCACTCTCAGTACAATCTGCTCTGA
TGCCGCATAGTTAAGCCAGCCCCGACACCCGCCAACACCCGCTGACGCGCCCTGACGGGC
TTGTCTGCTCCCGGCATCCGCTTACAGACAAGCTGTGACCGTCTCCGGGAGCTGCATGTGTCA
GAGGTTTTCACCGTCATCACCGAAACGCGCGAGACGAAAGGGCCTCGTGATACGCCTATTTTTA
TAGGTTAATGTCATGATAATAATGGTTTCTTAGACGTCAGGTGGCACTTTTCGGGGAAATGTGCGC
GGAACCCCTATTTGTTTATTTTTCTAAATACATTCAAATATGTATCCGCTCATGAGACAATAACCCTG
ATAAATGCTTCAATAATATTGAAAAAGGAAGAGTATGAGTATTCAACATTTCCGTGTCGCCCTTATTC
CCTTTTTTGCGGCATTTTGCCTTCCTGTTTTTGCTCACCCAGAAACGCTGGTGAAAGTAAAAGATG
CTGAAGATCAGTTGGGTGCACGAGTGGGTTACATCGAACTGGATCTCAACAGCGGTAAGATCCT
TGAGAGTTTTCGCCCCGAAGAACGTTTTCCAATGATGAGCACTTTTAAAGTTCTGCTATGTGGCGC
GGTATTATCCCGTATTGACGCCGGGCAAGAGCAACTCGGTCGCCGCATACACTATTCTCAGAAT
GACTTGGTTGAGTACTCACCAGTCACAGAAAAGCATCTTACGGATGGCATGACAGTAAGAGAATT
ATGCAGTGCTGCCATAACCATGAGTGATAACACTGCGGCCAACTTACTTCTGACAACGATCGGA
GGACCGAAGGAGCTAACCGCTTTTTTGCACAACATGGGGGATCATGTAACTCGCCTTGATCGTT
GGGAACCGGAGCTGAATGAAGCCATACCAAACGACGAGCGTGACACCACGATGCCTGTAGCA

ATGGCAACAACGTTGCGCAAACTATTAACTGGCGAACTACTTACTCTAGCTTCCCGGCAACAATT
AATAGACTGGATGGAGGCGGATAAAGTTGCAGGACCACTTCTGCGCTCGGCCCTTCCGGCTGG
CTGGTTTATTGCTGATAAATCTGGAGCCGGTGAGCGTGGGTCTCGCGGTATCATTGCAGCACTG
GGGCCAGATGGTAAGCCCTCCCGTATCGTAGTTATCTACACGACGGGGAGTCAGGCAACTATG
GATGAACGAAATAGACAGATCGCTGAGATAGGTGCCTCACTGATTAAGCATTGGTAACTGTCAGA
CCAAGTTTACTCATATATACTTTAGATTGATTTAAAACTTCATTTTTAATTTAAAAGGATCTAGGTGAAG
ATCCTTTTTGATAATCTCATGACCAAAATCCCTTAACGTGAGTTTTCGTTCCACTGAGCGTCAGAC
CCCGTAGAAAAGATCAAAGGATCTTCTTGAGATCCTTTTTTTCTGCGCGTAATCTGCTGCTTGCAA
ACAAAAAAACCACCGCTACCAGCGGTGGTTTGTTTGCCGGATCAAGAGCTACCAACTCTTTTTC
CGAAGGTAACTGGCTTCAGCAGAGCGCAGATACCAAATACTGTTCTTCTAGTGTAGCCGTAGTTA
GGCCACCACTTCAAGAACTCTGTAGCACCGCCTACATACCTCGCTCTGCTAATCCTGTTACCAG
TGGCTGCTGCCAGTGGCGATAAGTCGTGTCTTACCGGGTTGGACTCAAGACGATAGTTACCGGA
TAAGGCGCAGCGGTCGGGCTGAACGGGGGGTTCGTGCACACAGCCCAGCTTGGAGCGAAC
GACCTACACCGAACTGAGATACCTACAGCGTGAGCTATGAGAAAGCGCCACGCTTCCCGAAG
GGAGAAAGGCGGACAGGTATCCGGTAAGCGGCAGGGTCGGAACAGGAGAGCGCACGAGGG
AGCTTCCAGGGGGAAACGCCTGGTATCTTTATAGTCCTGTCGGGTTTCGCCACCTCTGACTTGA
GCGTCGATTTTTGTGATGCTCGTCAGGGGGGCGGAGCCTATGGAAAAACGCCAGCAACGCGG
CCTTTTTACGGTTCCTGGCCTTTTGCTGGCCTTTTGCTCACATGTTCTTTCCTGCGTTATCCCCTGA
TTCTGTGGATAACCGTATTACCGCCTTTGAGTGAGCTGATACCGCTCGCCGCAGCCGAACGAC
CGAGCGCAGCGAGTCAGTGAGCGAGGAAGCGGAAGAGCGCCCAATACGCAAACCGCCTCT
CCCCGCGCGTTGGCCGATTCATTAATGCAGCTGTGGAATGTGTGTCAGTTAGGGTGTGGAAAGT
CCCCAGGCTCCCCAGCAGGCAGAAGTATGCAAAGCATGCATCTCAATTAGTCAGCAACCAGGT
GTGGAAAGTCCCCAGGCTCCCCAGCAGGCAGAAGTATGCAAAGCATGCATCTCAATTAGTCAG
CAACCATAGTCCCGCCCCTAACTCCGCCCATCCCGCCCCTAACTCCGCCCAGTTCCGCCCAT
TCTCCGCCCCATGGCTGACTAATTTTTTTTATTTATGCAGAGGCCGAGGCCGCCTCGGCCTCTG
AGCTATTCCAGAAGTAGTGAGGAGGCTTTTTTGGAGGCCTAGGCTTTTGCAAAAAGCTTGGACAC
AAGACAGGCTTGCGAGATATGTTTGAGAATACCACTTTATCCCGCGTCAGGGAGAGGCAGTGCG
TAAAAAGACGCGGACTCATGTGAAATACTGGTTTTTAGTGCGCCAGATCTCTATAATCTCGCGCAA
CCTATTTTCCCCTCGAACACTTTTTAAGCCGTAGATAAACAGGCTGGGACACTTCACATGAGCGA
AAAATACATCGTCACCTGGGACATGTTGCAGATCCATGCACGTAAACTCGCAAGCCGACTGATG
CCTTCTGAACAATGGAAAGGCATTATTGCCGTAAGCCGTGGCGGTCTGTACCGGGTGCGTTACT
GGCGCGTGAACTGGGTATTCGTCATGTCGATACCGTTTGTATTTCCAGCTACGATCACGACAACC
AGCGCGAGCTTAAAGTGCTGAAACGCGCAGAAGGCGATGGCGAAGGCTTCATCGTTATTGATG
ACCTGGTGGATACCGGTGGTACTGCGGTTGCGATTCGTGAAATGTATCCAAAAGCGCACTTTGTC
ACCATCTTCGCAAAACCGGCTGGTCGTCCGCTGGTTGATGACTATGTTGTTGATATCCCGCAAGA
TACCTGGATTGAACAGCCGTGGGATATGGGCGTCGTATTCGTCCCGCCAATCTCCGGTCGCTAA
TCTTTTCAACGCCTGGCACTGCCGGGCGTTGTTCTTTTTAACTTCAGGCGGGTTACAATAGTTTCC
AGTAAGTATTCTGGAGGCTGCATCCATGACACAGGCAAACCTGAGCGAAACCCTGTTCAAACCC

CGCTTTAAACATCCTGAAACCTCGACGCTAGTCCGCCGCTTTAATCACGGCGCACAACCGCCT
GTGCAGTCGGCCCTTGATGGTAAAACCATCCCTCACTGGTATCGCATGATTAACCGTCTGATGTG
GATCTGGCGCGGCATTGACCCACGCGAAATCCTCGACGTCCAGGCACGTATTGTGATGAGCGA
TGCCGAACGTACCGACGATGATTTATACGATACGGTGATTGGCTACCGTGGCGGCAACTGGATT
TATGAGTGGGCCCCGGATCTTTGTGAAGGAACCTTACTTCTGTGGTGTGACATAATTGGACAAACT
ACCTACAGAGATTTAAAGCTCTAAGGTAAATATAAAATTTTTAAGTGTATAATGTGTTAAACTACTGATT
CTAATTGTTTGTGTATTTTAGATTCCAACCTATGGAACTGATGAATGGGAGCAGTGGTGGAATGCCT
TTAATGAGGAAAACCTGTTTTGCTCAGAAGAAATGCCATCTAGTGATGATGAGGCTACTGCTGACT
CTCAACATTCTACTCCTCCAAAAAAGAAGAGAAAGGTAGAAGACCCCAAGGACTTTCCTTCAGA
ATTGCTAAGTTTTTTGAGTCATGCTGTGTTTAGTAATAGAACTCTTGCTTGCTTTGCTATTTACACCAC
AAAGGAAAAAGCTGCACTGCTATACAAGAAAATTATGGAAAAATATTCTGTAACCTTTATAAGTAGG
CATAACAGTTATAATCATAACATACTGTTTTTTCTTACTCCACACAGGCATAGAGTGTCTGCTATTAAT
AACTATGCTCAAAAATTGTGTACCTTTAGCTTTTTAATTTGTAAAGGGGTTAATAAGGAATATTTGATG
TATAGTGCCTTGACTAGAGATCATAATCAGCCATACCACATTTGTAGAGGTTTTACTTGCTTTAAAAA
ACCTCCCACACCTCCCCCTGAACCTGAAACATAAAATGAATGCAATTGTTGTTGTTAACTTGTTTAT
TGCAGCTTATAATGGTTACAAATAAAGCAATAGCATCACAAATTTCACAAATAAAGCATTTTTTTCAC
TGCATTCTAGTTGTGGTTTGTCCAAACTCATCAATGTATCTTATCATGTCTGGATCAACTGGATAACT
CAAGCTAACCAAAATCATCCCAAACTTCCCACCCCATACCCTATTACCACTGCCAATTACCTGTG
GTTTCATTTACTCTAAACCTGTGATTCCTCTGAATTATTTTCATTTTAAAGAAATTGTATTTGTTAAATAT
GTACTACAAACTTAGTAGTTGGAAGGGCTAATTCACTCCCAAAGAAGACAAGATATCCTTGATCTG
TGGATCTACCACACACAAGGCTACTTCCCTGATTAGCAGAACTACACACCAGGGCCAGGGGTC
AGATATCCACTGACCTTTGGATGGTGCTACAAGCTAGTACCAGTTGAGCCAGATAAGGTAGAAGA
GGCCAATAAAGGAGAGAACACCAGCTTGTTACACCCTGTGAGCCTGCATGGGATGGATGACCC
GGAGAGAGAAGTGTTAGAGTGGAGGTTTGACAGCCGCCTAGCATTTCATCACGTGGCCCGAGA
GCTGCATCCGGAGTACTTCAAGAACTGCTGATATCGAGCTTGCTACAAGGGACTTTCCGCTGGG
GACTTTCCAGGGAGGCGTGGCCTGGGCGGGACTGGGGAGTGGCGAGCCCTCAGATCCTGCA
TATAAGCAGCTGCTTTTTGCCTGTACTGGGTCTCTCTGGTTAGACCAGATCTGAGCCTGGGAGCT
CTCTGGCTAACTAGGGAACCCACTGCTTAAGCCTCAATAAAGCTTGCCTTGAGTGCTTCAAGTAG
TGTGTGCCCGTCTGTTGTGTGACTCTGGTAACTAGAGATCCCTCAGACCCTTTTAGTCAGTGTGGA
AAATCTCTAGCAGTGGCGCCCGAACAGGGACTTGAAAGCGAAAGGGAAACCAGAGGAGCTCT
CTCGACGCAGGACTCGGCTTGCTGAAGCGCGCACGGCAAGAGGCGAGGGGCGGCGACTGG
TGAGTACGCCAAAAATTTTGACTAGCGGAGGCTAGAAGGAGAGAGATGGGTGCGAGAGCGTCA
GTATTAAGCGGGGGAGAATTAGATCGCGATGGGAAAAAATTCGGTTAAGGCCAGGGGGAAAGA
AAAAATATAAATTAAAACATATAGTATGGGCAAGCAGGGAGCTAGAACGATTCGCAGTTAATCCTG
GCCTGTTAGAAACATCAGAAGGCTGTAGACAAATACTGGGACAGCTACAACCATCCCTTCAGAC
AGGATCAGAAGAACTTAGATCATTATATAATACAGTAGCAACCCTCTATTGTGTGCATCAAAGGATA
GAGATAAAAGACACCAAGGAAGCTTTAGACAAGATAGAGGAAGAGCAAAACAAAAGTAAGACCA
CCGCACAGCAAGCGGCCGGCCGCTGATCTTCAGACCTGGAGGAGGAGATATGAGGGACAATT

GGAGAAGTGAATTATATAAATATAAAGTAGTAAAAATTGAACCATTAGGAGTAGCACCCACCAAGG
CAAAGAGAAGAGTGGTGCAGAGAGAAAAAAGAGCAGTGGGAATAGGAGCTTTGTTCCTTGGGTT
CTTGGGAGCAGCAGGAAGCACTATGGGCGCAGCGTCAATGACGCTGACGGTACAGGCCAGA
CAATTATTGTCTGGTATAGTGCAGCAGCAGAACAATTTGCTGAGGGCTATTGAGGCGCAACAGCA
TCTGTTGCAACTCACAGTCTGGGGCATCAAGCAGCTCCAGGCAAGAATCCTGGCTGTGGAAAG
ATACCTAAAGGATCAACAGCTCCTGGGGATTTGGGGTTGCTCTGGAAAACTCATTTGCACCACTG
CTGTGCCTTGGAATGCTAGTTGGAGTAATAAATCTCTGGAACAGATTTGGAATCACACGACCTGG
ATGGAGTGGGACAGAGAAATTAACAATTACACAAGCTTAATACACTCCTTAATTGAAGAATCGCAA
AACCAGCAAGAAAAGAATGAACAAGAATTATTGGAATTAGATAAATGGGCAAGTTTGTGGAATTGG
TTTAACATAACAAATTGGCTGTGGTATATAAAATTATTCATAATGATAGTAGGAGGCTTGGTAGGTTTA
AGAATAGTTTTTGCTGTACTTTCTATAGTGAATAGAGTTAGGCAGGGATATTCACCATTATCGTTTCA
GACCCACCTCCCAACCCCGAGGGGACCCGACAGGCCCGAAGGAATAGAAGAAGAAGGTGG
AGAGAGAGACAGAGACAGATCCATTCGATTAGTGAACGGATCTCGACGGTATCGCCAAATGGCA
GTATTCATCCACAATTTTAAAAGAAAAGGGGGGATTGGGGGGTACAGTGCAGGGGAAAGAATAG
TAGACATAATAGCAACAGACATACAAACTAAAGAATTACAAAAACAAATTACAAAAATTCAAAATTTT
CGGGTTTATTACAGGGACAGCAGAGATCCAGTTTGGATCGATAAGCTTGATATCGAATTCCTGCA
GCCCCGATAAAATAAAAGATTTTATTTAGTCTCCAGAAAAAGGGGGGAATGAAAGACCCCACCTG
TAGGTTTGGCAAGCTAGCTGCAGTAACGCCATTTTGCAAGGCATGGAAAAATACCAAACCAAGA
ATAGAGAAGTTCAGATCAAGGGCGGGTACATGAAAATAGCTAACGTTGGGCCAAACAGGATATCT
GCGGTGAGCAGTTTCGGCCCCGGCCCGGGGCCAAGAACAGATGGTCACCGCAGTTTCGGC
CCCGGCCCGAGGCCAAGAACAGATGGTCCCCAGATATGGCCCAACCCTCAGCAGTTTCTTAA
GACCCATCAGATGTTTCCAGGCTCCCCCAAGGACCTGAAATGACCCTGCGCCTTATTTGAATTA
ACCAATCAGCCTGCTTCTCGCTTCTGTTCGCGCGCTTCTGCTTCCCGAGCTCTATAAAAGAGCT
CACAACCCCTCACTCGGCGCGCCAGTCCTCCGACAGACTGAGTCGCCCGGGGGGGATCCA
CCGGTCGCCACCATGAGAGGCGCTCGCGGCGCCTGGGATTTTCTCTGCGTTCTGCTCCTACT
GCTTCGCGTCCAGACAGGCTCTTCTCAACCATCTGTGAGTCCAGGGGAACCGTCTCCACCATC
CATCCATCCAGGAAAATCAGACTTAATAGTCCGCGTGGGCGACGAGATTAGGCTGTTATGCACT
GATCCGGGCTTTGTCAAATGGACTTTTGAGATCCTGGATGAAACGAATGAGAATAAGCAGAATGA
ATGGATCACGGAAAAGGCAGAAGCCACCAACACCGGCAAATACACGTGCACCAACAAACACG
GCTTAAGCAATTCCATTTATGTGTTTGTTAGAGATCCTGCCAAGCTTTTCCTTGTTGACCGCTCCTT
GTATGGGAAAGAAGACAACGACACGCTGGTCCGCTGTCCTCTCACAGACCCAGAAGTGACCAA
TTATTCCCTCAAGGGGTGCCAGGGGAAGCCTCTTCCCAAGGACTTGAGGTTTATTCCTGACCCC
AAGGCGGGCATCATGATCAAAAGTGTGAAACGCGCCTACCATCGGCTCTGTCTGCATTGTTCTG
TGGACCAGGAGGGCAAGTCAGTGCTGTCGGAAAAATTCATCCTGAAAGTGAGGCCAGCCTTCA
AAGCTGTGCCTGTTGTGTCTGTGTCCAAAGCAAGCTATCTTCTTAGGGAAGGGGAAGAATTCACA
GTGACGTGCACAATAAAAGATGTGTCTAGTTCTGTGTACTCAACGTGGAAAAGAGAAAACAGTCA
GACTAAACTACAGGAGAAATATAATAGCTGGCATCACGGTGACTTCAATTATGAACGTCAGGCAA
CGTTGACTATCAGTTCAGCGAGAGTTAATGATTCTGGAGTGTTCATGTGTTATGCCAATAATACTTTT

GGATCAGCAAATGTCACAACAACCTTGGAAGTAGTAGATAAAGGATTCATTAATATCTTCCCCATG
ATAAACACTACAGTATTTGTAAACGATGGAGAAAATGTAGATTTGATTGTTGAATATGAAGCATTCCC
CAAACCTGAACACCAGCAGTGGATCTATATGAACAGAACCTTCACTGATAAATGGGAAGATTATC
CCAAGTCTGAGAATGAAAGTAATATCAGATACGTAAGTGAACTTCATCTAACGAGATTAAAAGGCA
CCGAAGGAGGCACTTACACATTCCTAGTGTCCAATTCTGACGTCAATGCTGCCATAGCATTTAAT
GTTTATGTGAATACAAAACCAGAAATCCTGACTTACGACAGGCTCGTGAATGGCATGCTCCAATGT
GTGGCAGCAGGATTCCCAGAGCCCACAATAGATTGGTATTTTTGTCCAGGAACTGAGCAGAGAT
GCTCTGCTTCTGTACTGCCAGTGGATGTGCAGACACTAAACTCATCTGGGCCACCGTTTGGAAA
GCTAGTGGTTCAGAGTTCTATAGATTCTAGTGCATTCAAGCACAATGGCACGGTTGAATGTAAGGC
TTACAACGATGTGGGCAAGACTTCTGCCTATTTTAACTTTGCATTTAAAGAGCAAATCCATCCCCA
CACCCTGTTCACTCCTTTGCTGATTGGTTTCGTAATCGTAGCTGGCATGATGTGCATTATTGTGATG
ATTCTGACCTACAAATATTTACAGAAACCCATGTATGAAGTACAGTGGAAGGTTGTTGAGGAGATAA
ATGGAAACAATTATGTTTACATAGACCCAACACAACTTCCTTATGATCACAAATGGGAGTTTCCCA
GAAACAGGCTGAGTTTTGGGAAAACCCTGGGTGCTGGAGCTTTCGGGAAGGTTGTTGAGGCAA
CTGCTTATGGCTTAATTAAGTCAGATGCGGCCATGACTGTCGCTGTAAAGATGCTCAAGCCGAGT
GCCCATTTGACAGAACGGGAAGCCCTCATGTCTGAACTCAAAGTCCTGAGTTACCTTGGTAATCA
CATGAATATTGTGAATCTACTTGGAGCCTGCACCATTGGAGGGCCCACCCTGGTCATTACAGAAT
ATTGTTGCTATGGTGATCTTTTGAATTTTTTGAGAAGAAAACGTGATTCATTTATTTGTTCAAAGCAGG
AAGATCATGCAGAAGCTGCACTTTATAAGAATCTTCTGCATTCAAAGGAGTCTTCCTGCAGCGATA
GTACTAATGAGTACATGGACATGAAACCTGGAGTTTCTTATGTTGTCCCAACCAAGGCCGACAAA
AGGAGATCTGTGAGAATAGGCTCATACATAGAAAGAGATGTGACTCCCGCCATCATGGAGGATGA
CGAGTTGGCCCTAGACTTAGAAGACTTGCTGAGCTTTTCTTACCAGGTGGCAAAGGGCATGGCT
TTCCTCGCCTCCAAGAATTGTATTCACAGAGACTTGGCAGCCAGAAATATCCTCCTTACTCATGGT
CGGATCACAAAGATTTGTGATTTTGGTCTAGCCAGAGACATCAAGAATGATTCTAATTATGTGGTTA
AAGGAAACGCTCGACTACCTGTGAAGTGGATGGCACCTGAAAGCATTTTCAACTGTGTATACACG
TTTGAAAGTGACGTCTGGTCCTATGGGATTTTTCTTTGGGAGCTGTTCTCTTTAGGAAGCAGCCCC
TATCCTGGAATGCCGGTCGATTCTAAGTTCTACAAGATGATCAAGGAAGGCTTCCGGATGCTCAG
CCCTGAACACGCACCTGCTGAAATGTATGACATAATGAAGACTTGCTGGGATGCAGATCCCCTA
AAAAGACCAACATTCAAGCAAATTGTTCAGCTAATTGAGAAGCAGATTTCAGAGAGCACCAATCA
TATTTACTCCAACTTAGCAAACTGCAGCCCCAACCGACAGAAGCCCGTGGTAGACCATTCTGTG
CGGATCAATTCTGTCGGCAGCACCGCTTCCTCCTCCCAGCCTCTGCTTGTGCACGACGATGTC
TGAAGCGGCC
